# DisConST: Distribution-aware Contrastive Learning for Spatial Domain Identification

**DOI:** 10.1093/gpbjnl/qzaf085

**Published:** 2025-09-24

**Authors:** Peimeng Zhen, Xiaofeng Wang, Han Shu, Jialu Hu, Yongtian Wang, Jiajie Peng, Xuequn Shang, Jing Chen, Tao Wang

**Affiliations:** School of Computer Science, Northwestern Polytechnical University, Xi’an 710072, China; Key Laboratory of Big Data Storage and Management, Ministry of Industry and Information Technology, Northwestern Polytechnical University, Xi’an 710072, China; Department of General Surgery, The Affiliated Hospital of Northwest University, Xi’an No. 3 Hospital, Xi’an 710018, China; School of Computer Science, Northwestern Polytechnical University, Xi’an 710072, China; Key Laboratory of Big Data Storage and Management, Ministry of Industry and Information Technology, Northwestern Polytechnical University, Xi’an 710072, China; School of Computer Science, Northwestern Polytechnical University, Xi’an 710072, China; Key Laboratory of Big Data Storage and Management, Ministry of Industry and Information Technology, Northwestern Polytechnical University, Xi’an 710072, China; School of Computer Science, Northwestern Polytechnical University, Xi’an 710072, China; Key Laboratory of Big Data Storage and Management, Ministry of Industry and Information Technology, Northwestern Polytechnical University, Xi’an 710072, China; School of Computer Science, Northwestern Polytechnical University, Xi’an 710072, China; Key Laboratory of Big Data Storage and Management, Ministry of Industry and Information Technology, Northwestern Polytechnical University, Xi’an 710072, China; School of Computer Science, Northwestern Polytechnical University, Xi’an 710072, China; Key Laboratory of Big Data Storage and Management, Ministry of Industry and Information Technology, Northwestern Polytechnical University, Xi’an 710072, China; School of Computer Science and Engineering, Xi’an University of Technology, Xi’an 710048, China; School of Computer Science, Northwestern Polytechnical University, Xi’an 710072, China; Key Laboratory of Big Data Storage and Management, Ministry of Industry and Information Technology, Northwestern Polytechnical University, Xi’an 710072, China

**Keywords:** Spatial transcriptomics, Spatial domain identification, Zero-inflated negative binomial distribution, Graph contrastive learning, Graph neural network

## Abstract

Spatial transcriptomics (ST) is a cutting-edge technology that provides comprehensive insights into gene expression (GE) patterns from a spatial perspective. A key research focus within this field is spatial domain identification, which is essential for exploring tissue organization, biological development, and disease mechanisms. Although methods have been developed, they still face challenges in modeling GE information together with spatial locations, resulting in suboptimal accuracy. Here, we introduce Distribution-aware Contrastive Learning for Spatial Transcriptomics (DisConST), a novel deep learning method designed to improve spatial domain detection within ST datasets. DisConST addresses key challenges, such as the high dropout rates and the complex integration of spatial and GE data, by incorporating contrastive learning strategies that are aware of the underlying data distributions. It employs the zero-inflated negative binomial distribution, along with graph contrastive learning, to generate more informative latent representations. These representations efficiently integrate spatial positions, transcriptomic profiles, and cell-type proportions within spots. We validated DisConST across diverse ST datasets of tissues, organs, and embryos from various sequencing platforms in both normal and disease states. Our results consistently demonstrate that DisConST achieves superior spatial domain recognition accuracy compared to existing state-of-the-art methods. Furthermore, our experiments highlight the utility of DisConST in advancing research on tissue organization, embryonic development, and tumor immune microenvironment dissection. The source code for DisConST is freely available at https://github.com/Zhenpm/DisConST/.

## Graphical abstract

**Figure:**
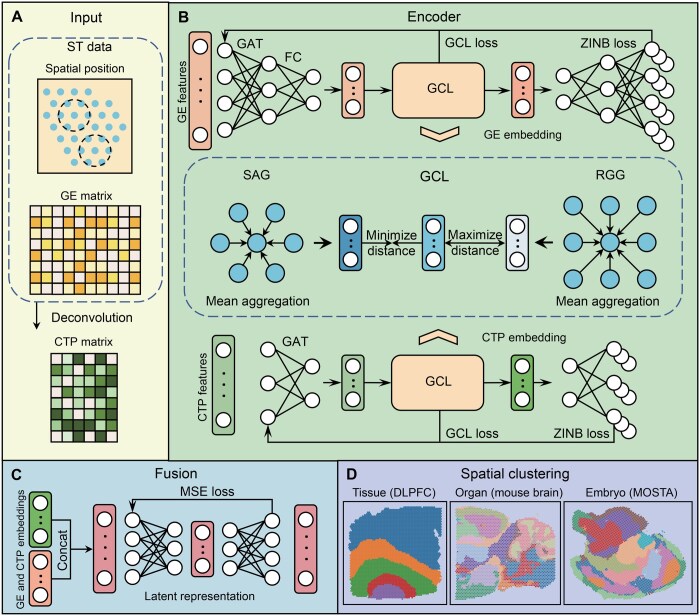

## Introduction

The spatial organization of cells within tissues is a fundamental aspect of biological systems, influencing everything from developmental processes to disease progression. In tissues such as embryos and tumors, cellular interactions and spatial context play pivotal roles in function and behavior [1]. While conventional single-cell transcriptomics has provided unprecedented insights into cellular gene expression (GE), it falls short of capturing spatial relationships between cells, limiting our understanding of tissue architecture and function [2–5]. Spatial transcriptomics (ST) is an emerging technology designed to bridge this gap by enabling the comprehensive mapping of GE profiles across tissue sections while preserving spatial information [6–8]. This advancement has opened new avenues for studying cellular organization, tissue structure, and functional heterogeneity in various biological systems. However, a critical task in ST research is the identification of spatial domains — regions within tissues that exhibit similar GE patterns and coherent spatial properties.

ST techniques are broadly divided into two categories: next-generation sequencing (NGS)-based and imaging-based methods. NGS-based methods, such as 10x Visium [9], generate spatial GE profiles by sequencing transcripts from distinct tissue spots. Imaging-based techniques, such as *in situ* hybridization (ISH) and *in situ* sequencing (ISS), offer higher spatial resolution by directly visualizing and quantifying transcripts within tissue sections [10,11]. Each approach provides distinct advantages in resolution and throughput, but both share the challenge of identifying meaningful spatial domains from high-dimensional and often noisy data.

Traditional clustering algorithms, such as K-means and Louvain, have been applied to categorize spatial domains based solely on GE. However, these methods often overlook critical spatial features, leading to discontinuous or inaccurate domain structures. To address these limitations, several advanced techniques have been developed. For example, stLearn [12] normalizes GE data using neighborhood information and morphological distance to identify domain structures. Spatially Embedded Deep Representation (SEDR) [13] combines deep autoencoders with masked self-supervised learning to create low-dimensional latent representations of GE, embedding spatial information with a variational graph autoencoder for domain identification. SpaGCN [14] enhances clustering performance by integrating histological and spatial information, while Cell Clustering for Spatial Transcriptomics (CCST) [15] leverages contrastive learning through randomly constructed graphs to optimize model parameters. BayesSpace [16] employs Bayesian clustering to assign higher weights to physically proximate points, improving model refinement. Similarly, STAGATE [17] utilizes a graph attention (GAT) encoder to merge spatial and GE information, while GraphST [18] enhances representational power through random shuffling of GE profiles and contrastive learning. Despite these advancements, existing methods still face challenges due to the high dropout rates in GE profiles typical of ST datasets and difficulties in effectively integrating spatial information, making it challenging to generate highly informative latent representations for accurate domain identification.

To overcome these challenges, we propose Distribution-aware Contrastive Learning for Spatial Transcriptomics (DisConST), a novel method designed to enhance spatial domain detection by leveraging both ST data and supplementary cell-type proportion (CTP) information. DisConST employs a deep learning framework, using the zero-inflated negative binomial (ZINB) distribution to model GE, which accounts for the sparsity and overdispersion typical of biological data [19–22]. In addition, DisConST integrates a contrastive learning strategy, optimizing latent representations by aligning GE and spatial features through the generation of random graphs and adjacency graphs. These representations are further refined by fusing GE data and CTPs through a fully connected (FC) encoder, yielding a highly informative latent space for accurate spatial domain detection. We evaluated the performance of DisConST across multiple ST datasets from both normal and diseased tissues, including organs and embryonic tissues. Our results demonstrate that DisConST consistently outperforms existing state-of-the-art methods in spatial domain identification, providing more accurate and robust insights into tissue organization and function.

## Method

**Figure 1:**
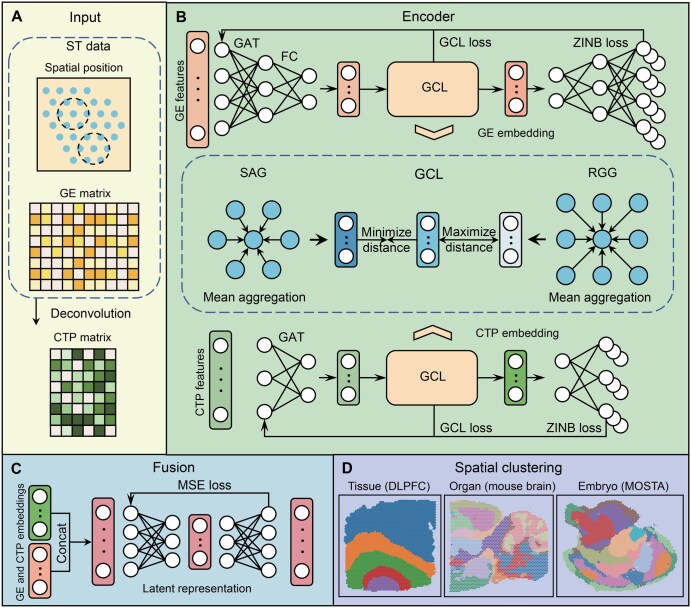
Overview of DisConST workflow **A**. Input of DisConST. DisConST processes three types of input data: GE data, spatial coordinates, and CTP data. It constructs an SAG by incorporating spatial information from the ST data. The CTPs are derived using deconvolution method. **B**. Encoder architecture of DisConST. DisConST uses a dual-encoder architecture to encode both the GE data and CTP data with a similar structure. The encoder is optimized with a combination of ZINB loss and GCL loss. **C**. Representation fusion. DisConST integrates the GE and CTP embeddings through an FC encoder to generate the final latent representation by concatenating these features. **D**. Spatial clustering for domain identification. DisConST applies spatial clustering techniques to recognize spatial domains in tissues, organs, and embryos. GE, gene expression; CTP, cell-type proportion; DisConST, Distribution-aware Contrastive Learning for Spatial Transcriptomics; GAT, graph attention; FC, fully connected; ST, spatial transcriptomics; GCL, graph contrastive learning; ZINB, zero-inflated negative binomial; SAG, spatial adjacency graph; RGG, randomly generated graph; MSE, mean squared error; DLPFC, dorsolateral prefrontal cortex; MOSTA, Mouse Organogenesis Spatiotemporal Transcriptomic Atlas.

This study presents DisConST, a deep learning method that effectively combines ST data with CTP data obtained through deconvolution to accurately identify spatial domains. The architecture of DisConST is organized into three main steps, as illustrated in **Figure 1**. First, DisConST employs an autoencoder optimized with the ZINB distribution and graph contrastive learning (GCL) to encode GE data and CTP data, respectively. The ZINB distribution is particularly suited for modeling the characteristics of GE and CTPs, while GCL enhances the discriminative power of the learned representations by leveraging positive and negative pairs. Second, DisConST integrates the GE and CTP representations obtained from the first step into a unified feature space using an FC encoder. Finally, DisConST utilizes a clustering algorithm to predict the spatial domains based on the fused representations.

### Data preprocessing

For the dataset used in this study, we first selected a set of highly variable genes (HVGs), typically set to 3000 for datasets with a sufficient number of sequenced genes. This selection was performed using the SCANPY package [23]. The selected genes underwent normalization and log transformation, which provided the initial features of GE for DisConST, referred to as GE features. Additionally, we processed the ST data using deconvolution tools such as SPACEL [24] and Tangram [25] to obtain CTPs for each spot. It should be noted that if the ST data are at single-cell resolution, the cell-type information is not considered. The log-transformed CTPs were then utilized as the second set of initial features in DisConST, termed CTP features.

### Spatial adjacency graph construction

The ST data provide the coordinates for each spot, which serve as the basis for constructing a spatial adjacency graph (SAG). In the SAG, each spot is represented as a node, with edges connecting nodes that are in close proximity. Specifically, we select the *k* nearest nodes for each node to construct the SAG. The specific selection of the number of neighbors *k* is shown in Table S1. We denote the adjacency matrix as A, where if there is an edge between nodes i and j, then $A_{ij}$ is set to 1; otherwise, $A_{ij}$ is set to 0.

### Graph encoder

To obtain the latent representation of spots, DisConST utilizes the SAG, along with the preprocessed GE matrix and CTP matrix, as inputs to the graph encoder. For encoding the GE data, we employ a two-layer neural network, where the first layer is a GAT layer [26], followed by an FC layer. The GAT layer incorporates an attention mechanism that adaptively assigns weights to a node’s neighbors, enhancing feature aggregation by emphasizing contributions from similar neighbors while diminishing those from dissimilar ones. The computation of attention weights occurs in two steps, beginning with the calculation of attention scores for the node pair (i,j) as follows:


(1) eij=δ(VsT(WGATXi)+VrT(WGATXj))

where $X_{i}$ and $X_{j}$ are the initial representations of nodes *i* and *j*, specifically, the input GE data. $V_{s}$ and $V_{r}$ are trainable parameters, while $W_{\mathrm{GAT}}$ serves as the trainable matrix for feature extraction in GAT. *δ* is the sigomid activation function. The second step is to normalize the attention score to obtain the attention coefficient, which can be described as follows:


(2) αij=softmax(eij)=exp⁡(eij)∑k∈N(i)exp⁡(eik)

where $\alpha_{ij}$ is the attention coefficient of node pair (i,j). N(i) is the set of all neighbors of node i. Based on the attention coefficient, GAT aggregates the representation of the central node from neighboring nodes:


(3) hi(GAT)=σ(∑j∈N(i)αijWGATXj)

where $h_{i}^{\left(\mathrm{GAT}\right)}$ represents the first layer representation of spot i obtained from the GAT layer, and σ is the Exponential Linear Unit (ELU) activation function. To further extract latent representations of GE features for each node, DisConST employs an FC layer to reduce the output dimension of the GAT layer:


(4) hi=σ(WFChi(GAT))

where $h_{i}$ represents the latent representation of spot i obtained from the FC layer, σ denotes the ELU activation function, and $W_{FC}$ is a trainable parameter.

For the CTP data, the encoding process is similar, but due to the relatively low number of cell types in many datasets, we use a single-layer GAT for encoding without a subsequent FC layer.

### Optimization strategy

The loss function of DisConST is divided into two parts: ZINB loss and GCL loss.

#### ZINB distribution-based loss

The ZINB distribution is commonly used to describe the characteristics of single-cell data [22]. Our experiments showed that the ZINB distribution performs well on ST data. The evidence lower bound (ELBO) is a loss function widely used in variational autoencoders (VAEs). It addresses the difficulty of directly computing the data distribution by maximizing the variational lower bound of the log-likelihood [27]. However, in the current context, we employ a graph autoencoder rather than a VAE, and face no challenges in computing data distribution. Therefore, we can directly optimize the parameters of the ZINB distribution via the negative log-likelihood function to achieve robust fitting of ST data. This distribution combines two probability distributions to better fit data with a large number of zeros. Its probability mass function can be expressed as follows:


(5) fZINB(X|μ,θ,π)=πδ0(X)+(1-π)fNB(X|μ,θ)

where π is the dropout probability (the proportion of zeros). $\delta_{0}$ is a one-point distribution that takes the value 1 at 0 and 0 at all other positions. $f_{NB}$ is the probability mass function of the negative binomial (NB) distribution, expressed as:


(6) fNB(X|μ,θ)=Γ(X+θ)X!Γ(θ)(θθ+μ)(μμ+θ)X

where μ and θ represent the mean and dispersion parameters of the NB distribution, respectively. To construct the ZINB loss, we design a special decoder to obtain the three parameters of the ZINB distribution. The decoder and encoder are symmetrical structures. For a two-layer encoder, we construct a two-layer decoder, with the first layer defined as follows:


(7) h^ij(GAT)=σ(∑j∈N(i)αijWGATThj)

Corresponding to the GAT layer, the first layer decoder uses the same parameters, where $W_{\mathrm{GAT}}^{T}$ is the transpose of $W_{\mathrm{GAT}}$ and ${\hat{h}}_{ij}^{\left(\mathrm{GAT}\right)}$ is the GAT decoding representation of node i. To obtain the three parameters of the ZINB distribution, the second layer has three parts, defined as follows:


(8) M=diag(si)⋅exp⁡(Wh^ij(GAT))

(9) Θ=exp⁡(Wh^ij(GAT))

(10) Π=δ(Wh^ij(GAT))

In the second layer, M, Θ, and Π represent matrix estimates of mean, dispersion, and dropout probability, respectively. For the mean and dispersion, we choose the exponential activation function, as both parameters are non-negative values. For the additional coefficient Π, the activation function δ is sigmoid, representing the dropout probability. As the dropout probability ranges from 0 to 1, sigmoid is an appropriate choice. W is a shared trainable parameter, and $s_{i}$ is the size factor of spot i, defined as:


(11) si=gimedian(gi)

where $g_{i}$ is the total GE of spot i, representing the relative GE of each spot. Similar to the encoder, for datasets with relatively few cell types, we also have only one decoder layer, specifically defined as follows:


(12) M=diag(si)⋅exp⁡(∑j∈N(i)WGATTh^ij(GAT))

(13) Θ=exp⁡(∑j∈N(i)WGATTh^ij(GAT))

(14) Π=δ(WGATTh^ij(GAT))

Finally, we use the negative log-likelihood function to calculate the ZINB loss. Specifically, the ZINB loss is defined as follows:


(15) LZINB=-log⁡(fZINB(X|μ,θ,π))

#### GCL-based loss

Although the graph autoencoder can integrate features from neighboring nodes to learn latent representations, it may lead to over-smoothing representations that obscure distinct biological features, complicating effective clustering. To enhance the discriminative power of the latent representation, we employ a GCL process.

Firstly, for each node i, we randomly select l nodes (excluding node i). Due to the significant gap between the total number of nodes in the graph and the number of neighbors of node i, these nodes are usually not neighbors of node i but rather located far from it. This gives us a randomly generated graph (RGG) $G^{\prime }=(V^{\prime }$, *E*′). We then aggregate the neighbors of each node in the SAG G and RGG $G^{'}$ through mean aggregation, defined as follows:


(16) hi(pos)=1n∑j∈N(i)hj

(17) hi(neg)=1n′∑k∈N′(i)hk

We denote $h_{i}$ as the representation of anchor spot i, where $h_{i}^{\left(\mathrm{pos}\right)}$ is the representation obtained by aggregating the neighbors of node i from the SAG, forming a positive pair with $h_{i}$, while $h_{i}^{\left(\mathrm{neg}\right)}$ is the representation obtained from the RGG, forming a negative pair with $h_{i}$. N(i) and $N^{\prime }(i)$ are the sets of all neighbors of node i in SAG and RGG, respectively, and n and $n^{\prime }$ are the numbers of neighbors corresponding to node i in the two graphs.

To achieve GCL by minimizing the Euclidean distance between positive pairs and maximizing the distance between negative pairs, the loss function is specifically formulated as follows:


(18) LGCL=1N∑i=1Nmax⁡(‖hi-hi(pos)‖2-‖hi-hi(neg)‖2+τ, 0)  

where τ is used to enforce a margin between the distances of positive and negative pairs, with a default value of 1. N represents the total number of spots, as each spot corresponds to one positive pair and one negative pair.

The final loss is represented as:


(19) L=LZINB+α⋅LGCL

where α is the coefficient for the GCL loss. The selection of α on the specific dataset is shown in Table S1.

### Feature fusion

To jointly utilize the latent representations obtained from GE data and CTP data for subsequent tasks, we employ an FC encoder to fuse the two representations as follows:


(20) zi=σ(Wfusehi(concat))

where $z_{i}$ represents the final latent representation of spot i, and $h_{i}^{\mathrm{concat}}$ is the representation obtained by concatenating the latent representations of GE and CTPs. Subsequently, DisConST utilizes an FC decoder to decode the fused representation. The fusion encoder employs mean squared error (MSE) loss to describe the reconstruction effect:


(21) Lfusion=1N‖h^i(concat)-hi(concat)‖2

where ${\hat{h}}_{i}^{\left(\mathrm{concat}\right)}$ represents the decoded reconstruction of $h_{i}^{\left(\mathrm{concat}\right)}$.

### Clustering and refinement

We default to utilizing the Gaussian mixture model from the mclust R package (v6.0.0) [28] to obtain clustering results for the latent representations learned by DisConST. The number of clusters is based on the ground truth labels. For data without a clear number of clusters, we recommend using the Louvain algorithm [29] for clustering. To enhance clustering accuracy, we implement a refinement step. For each spot, we define a radius (default value of 50) within which all spots are considered neighbors of spot. We then reassign spot to the domain with the most common label among its neighbors. Notably, this refinement process may not be suitable for fine-grained datasets, such as those from the mouse olfactory bulb (MOB), brain, and embryos. In this study, we applied this refinement step only to the human dorsolateral prefrontal cortex (DLPFC) and human breast cancer datasets.

### Benchmark

We compared DisConST with seven state-of-the-art methods, including stLearn [12], SEDR [13], SpaGCN [14], CCST [15], BayesSpace [16], STAGATE [17], and GraphST [18]. The introduction of these methods is shown in Table S2. The Adjusted Rand Index (ARI) [30] is used to evaluate the clustering results, defined as follows:


(22) ARI=2(ad-bc)(a+b)(b+d)+(a+c)(c+d)

In this equation, a denotes the number of spot pairs that are in the same cluster under both actual and experimental conditions. b represents the number of spot pairs that are in the same cluster in actual situations but not in the same cluster in experimental situations. c indicates the number of spot pairs that are not in the same cluster in actual situations but are in the same cluster in experimental situations. Finally, d stands for the number of spot pairs that are not in the same cluster in either actual or experimental situations. The ARI ranges from −1 to 1, with higher values signifying greater congruence between the clustering result and the ground truth labels, thereby indicating better clustering performance. On certain datasets, we additionally incorporate the Adjusted Mutual Information (AMI) [31], Normalized Mutual Information (NMI) [32], or Fowlkes–Mallows Index (FMI) [33] to provide a more comprehensive evaluation. We perform the following label conversion process before calculating the FMI metric. For each predicted cluster, we first filter out the spots belonging to that cluster and retrieve their corresponding true labels. Subsequently, we identify the most prevalent true class among them and designate it as the new label for the predicted cluster. This newly assigned label is then utilized to compute the FMI.

### Datasets

In our work, we employed six distinct datasets derived from various sequencing platforms, including 10x Visium, SpaTial Enhanced REsolution Omics-sequencing (Stereo-seq), Sequential Fluorescence *In Situ* Hybridization (seqFISH), and ST. The first dataset consists of 12 DLPFC tissue sections obtained from 10x Visium, with spot counts ranging between 3460 and 4789, encompassing 33,538 genes. These data, along with manual annotations, are available at http://research.libd.org/spatialLIBD/. The second dataset comprises MOB slices acquired with Stereo-seq and ST, with spot counts of 10,000 and 282, respectively. These ST data are available at https://github.com/hannshu/st_datasets. The third cohort includes mouse embryo slices derived from Stereo-seq and seqFISH (https://crukci.shinyapps.io/SpatialMouseAtlas/), containing 5913 and 19,416 spots, respectively. The fourth dataset includes four mouse brain tissue sections from the 10x Visium platform (https://www.10xgenomics.com/resources/datasets), exhibiting spot numbers ranging from 2696 to 3353. The fifth dataset includes four Mouse Organogenesis Spatiotemporal Transcriptomic Atlas (MOSTA) datasets with embryonic day 9.5 (E9.5) to E12.5 from the Stereo-seq platform (https://db.cngb.org/stomics/mosta/download/), with spot counts ranging from 5059 to 27,455. The last dataset features human breast cancer tissue samples from 10x Visium (https://www.10xgenomics.com/datasets/human-breast-cancer-block-a-section-1-1-standard-1-1-0 and https://www.10xgenomics.com/datasets/human-breast-cancer-ductal-carcinoma-in-situ-invasive-carcinoma-ffpe-1-standard-1-3-0), with spot counts of 3798 and 2518, respectively. A comprehensive summary of all these datasets can be found in Table S3.

For datasets from 10x Visium, where the resolution is multi-cell per spot, we used CTPs as supplementary data, necessitating the use of single-cell transcriptomics data for deconvolution. First, the single-nucleus transcriptomics data for human cortical areas is available at https://portal.brain-map.org/atlases-and-data/rnaseq/human-multiple-cortical-areas-smart-seq. Second, the human breast cancer single-cell transcriptomics data are available at https://singlecell.broadinstitute.org/single_cell/study/SCP1039. Finally, the single-cell transcriptomics data for the mouse whole brain are available at mousebrain.org/adolescent/downloads.html.

### Visualization and trajectory inference

The violin plots and box plots in this article are drawn by ggplot2 R package [34]. The Uniform Manifold Approximation and Projection (UMAP) [35] visualization plots and trajectory inference [36] visualization plots are drawn by SCANPY Python package [23].

## Results

### Overview of DisConST workflow

In this work, we propose a graph neural network-based method, DisConST, for representation learning and spatial domain identification of ST data (Figure 1). DisConST encodes the spot-level representations by considering both the GE and the cell types within a spot, simultaneously. Specifically, DisConST first constructs an SAG using the spatial locations of spots, and then utilizes a graph neural network to encode GE profiles and CTPs separately. The encoder consists of GAT and FC layers for feature extraction, which adaptively integrates features between the central spot and its spatially nearby spots. During the training process, the model is optimized by two strategies. First, the ZINB distribution is used in modeling the input features to address excess zeros and overdispersion simultaneously. Second, DisConST uses GCL to enhance the discrimination of the latent representations (see Method). After separately encoding GE and cell-type information, DisConST uses an additional FC layer to fuse the two representations. The final integrated representation is then used for spatial domain identification based on unsupervised clustering.

### DisConST enables accurate spatial domain identification on human DLPFC

In order to evaluate the performance of DisConST, we used 12 slices of the human DLPFC from 10x Visium sequencing technology for spatial domain identification [37]. The 12 slices of DLPFC have been manually annotated as 6 different layers and white matter (WM), which serve as the ground truth for evaluation. We compared DisConST with seven state-of-the-art methods, namely stLearn [12], SEDR [13], SpaGCN [14], CCST [15], BayesSpace [16], STAGATE [17], and GraphST [18]. Their performance was evaluated using the ARI metric [30].

**Figure 2:**
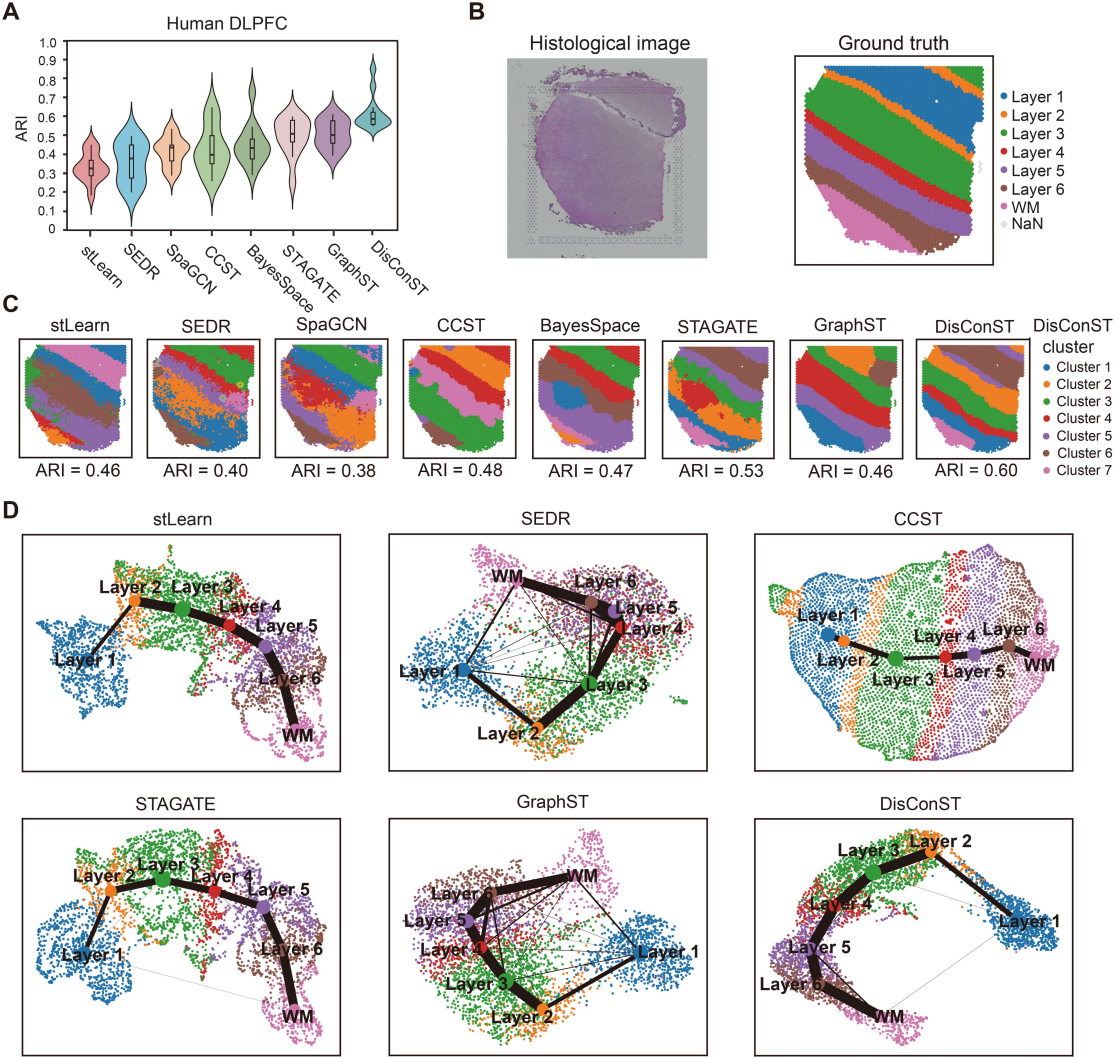
DisConST significantly improves the accuracy of spatial domain recognition on human DLPFC ST dataset **A**. The violin plot shows the ARI scores to summarize the accuracy of each method in all 12 slices of the DLPFC dataset. The box plot is embedded in the violin plot. The box plot’s center line, box limits, and whiskers denote the median, upper and lower quartiles, and 1.5× interquartile range, respectively. **B**. The histological image and ground truth with six cortex layers and WM of DLPFC slice #151507. **C**. Visualization of spatial domain detection of eight methods: stLearn, SEDR, SpaGCN, CCST, BayesSpace, STAGATE, GraphST, and DisConST on DLPFC slice #151507. **D**. UMAP plots and corresponding PAGA spots of latent representations obtained from six methods: stLearn, SEDR, CCST, STAGATE, GraphST, and DisConST. Notably, as end-to-end methods, SpaGCN and BayesSpace do not provide latent representations, making it impossible to visualize UMAP and PAGA plots. DLPFC, dorsolateral prefrontal cortex; ARI, Adjusted Rand Index; SEDR, spatially embedded deep representation; CCST, cell clustering for spatial transcriptomics; WM, white matter; NaN, not a number; UMAP, Uniform Manifold Approximation and Projection; PAGA, partition-based graph abstraction.

**Figure 2**A shows the performance of DisConST compared with seven state-of-the-art methods across 12 DLPFC slices. DisConST achieves an average ARI of 0.62, with a peak ARI of 0.86. Among the competitors, STAGATE and GraphST perform best, with average ARIs of 0.50 and 0.51, respectively. Additionally, we use the first slice (#151507) to further illustrate the clustering accuracy of DisConST compared to other methods (Figure 2B and C). Notably, only DisConST accurately captures the structure of layers 2 and 3, which are positioned on both sides of layer 1. Moreover, stLearn, SEDR, and SpaGCN fail to recognize the laminar structure from layers 4 to 6, resulting in relatively disorganized domain partitions. CCST incorrectly merges these three distinct layers into a single domain. While BayesSpace and STAGATE successfully identify the hierarchical arrangement of layers, they incorrectly produce an elliptical domain in this region. Lastly, GraphST erroneously splits layer 1 into two separate domains. In summary, DisConST is the only method that correctly identifies the overall layer structure. In addition to the comparison with ground truth, we further analyzed the spatial distribution of experimentally verified marker genes for identified cortex layers. As illustrated in Figure S1, the spatial distribution of marker genes aligns well with the layer domain that we have identified, which further demonstrates the accuracy of our method. A comprehensive summary of the performance of these methods in terms of ARI, AMI, and NMI can be found in Tables S4–S6. The visualization results of these methods on the 12 slices are shown in Figure S2.

Additionally, we generated UMAP visualization plots for DisConST and five other methods using DLPFC slice #151507 to further evaluate the expressiveness of their latent representations. The underlying assumption is that effective latent representations should accurately preserve the original relative positions of spatial domains. As shown in Figure 2D, the UMAP visualizations for SEDR and GraphST fail to capture the laminar structure among the six cortical layers and the WM, while other methods demonstrate clearer layer distribution patterns. Notably, compared to the other five methods, DisConST not only distinguishes each domain more effectively but also exhibits tighter clustering of spots within the same domain. To further validate the strength of our latent representations, we applied the partition-based graph abstraction (PAGA) algorithm [36] for trajectory inference. The trajectory plots are overlaid on the UMAP plots in Figure 2D. DisConST shows a nearly linear developmental trajectory from the first layer to the WM, with higher similarity between adjacent layers. In contrast, the trajectories inferred from SEDR and GraphST reveal little to no discernible linear relationships.

### DisConST demonstrates robust performance across different sequencing platforms

As ST technology evolves, various sequencing techniques with different resolution levels have emerged. To assess the performance of DisConST across different platforms, we conducted spatial domain identification using multiple sequencing technologies, including Stereo-seq, ST, and seqFISH. For this experiment, we used ST data from the MOB tissue sequenced by Stereo-seq [38] and ST [39]. Additionally, we analyzed developing mouse embryos using data from Stereo-seq [40] and seqFISH [41]. Both Stereo-seq and seqFISH offer single-cell resolution, whereas ST has a lower resolution, with each spot covering a diameter of approximately 100 µm.

**Figure 3:**
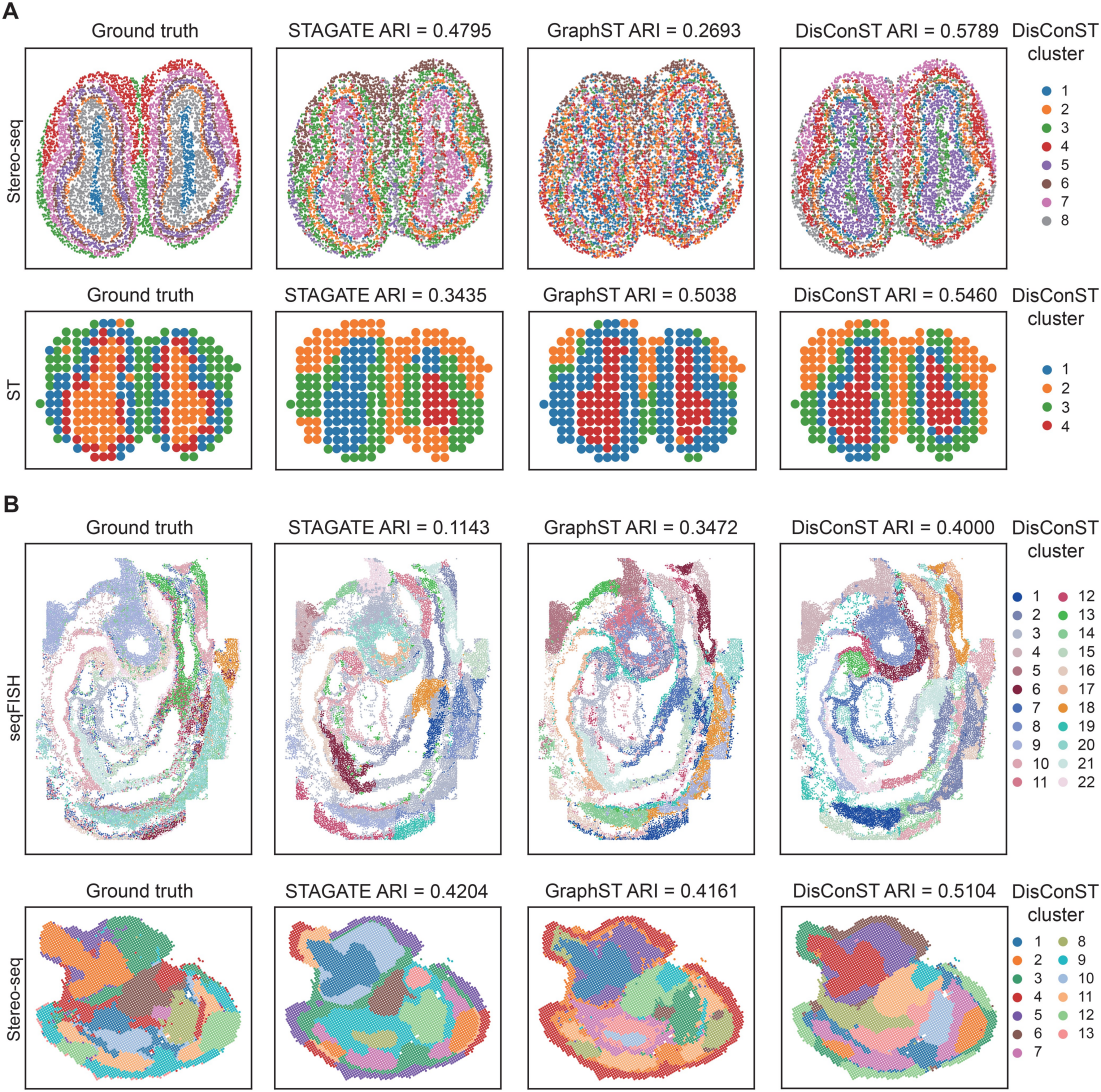
DisConST demonstrates robust performance across different sequencing platforms **A**. Ground truth and spatial domains identified by STAGATE, GraphST, and DisConST for the MOB tissue, sequenced using Stereo-seq and ST. **B**. Ground truth and spatial domains identified by STAGATE, GraphST, and DisConST for the MOSTA, sequenced using seqFISH and Stereo-seq. The ARI scores are indicated for each plot. Stereo-seq, SpaTial Enhanced REsolution Omics-sequencing; seqFISH, Sequential Fluorescence *In Situ* Hybridization; MOB, mouse olfactory bulb.

As shown in **Figure 3**A, DisConST significantly outperforms the two leading comparison models, STAGATE and GraphST, in identifying spatial domains within the MOB tissue using both the Stereo-seq and ST platforms. DisConST achieves an ARI above 0.5 for data from both platforms, while GraphST struggles to identify domain structures, resulting in mixed domain distributions. Additionally, as shown in Figure S3, DisConST more effectively reveals the laminar structure of the MOB compared to STAGATE and GraphST. Several marker genes have also validated the precision of our results. For example, *Apod* [42] has been demonstrated to be correlated with the olfactory nerve layer, and *Cck* [43] has been ascertained to be concentrated within the external plexiform layer. In addition, several other marker genes have been demonstrated to have functional implications in the MOB, such as *Doc2g* [44], *Bc1* [45], *Ptgds* [46], and *Nrxn3* [47]. To further validate DisConST’s cross-platform capability, we extended our experiments to developing mouse embryos, applying three clustering methods to seqFISH data from stages E8.5 to E8.75, and to Stereo-seq data from stage E9.5. Figure 3B demonstrates that, despite variations in developmental stages and tissue sequencing techniques, DisConST consistently outperforms the other two methods. To further evaluate the cross-platform performance, we compared DisConST with SEDR, SpaGCN, and CCST. As shown in Figure S4 and Table S7, SEDR and CCST show suboptimal performance on MOB data from ST platform. SpaGCN can basically identify the laminar structure of MOB and also reveal embryonic organs, but the ARI scores of SpaGCN are lower than DisConST. In summary, DisConST maintains robust spatial domain detection performance across different resolutions and sequencing platforms.

### DisConST effectively identifies the organizational structure of the mouse brain

**Figure 4:**
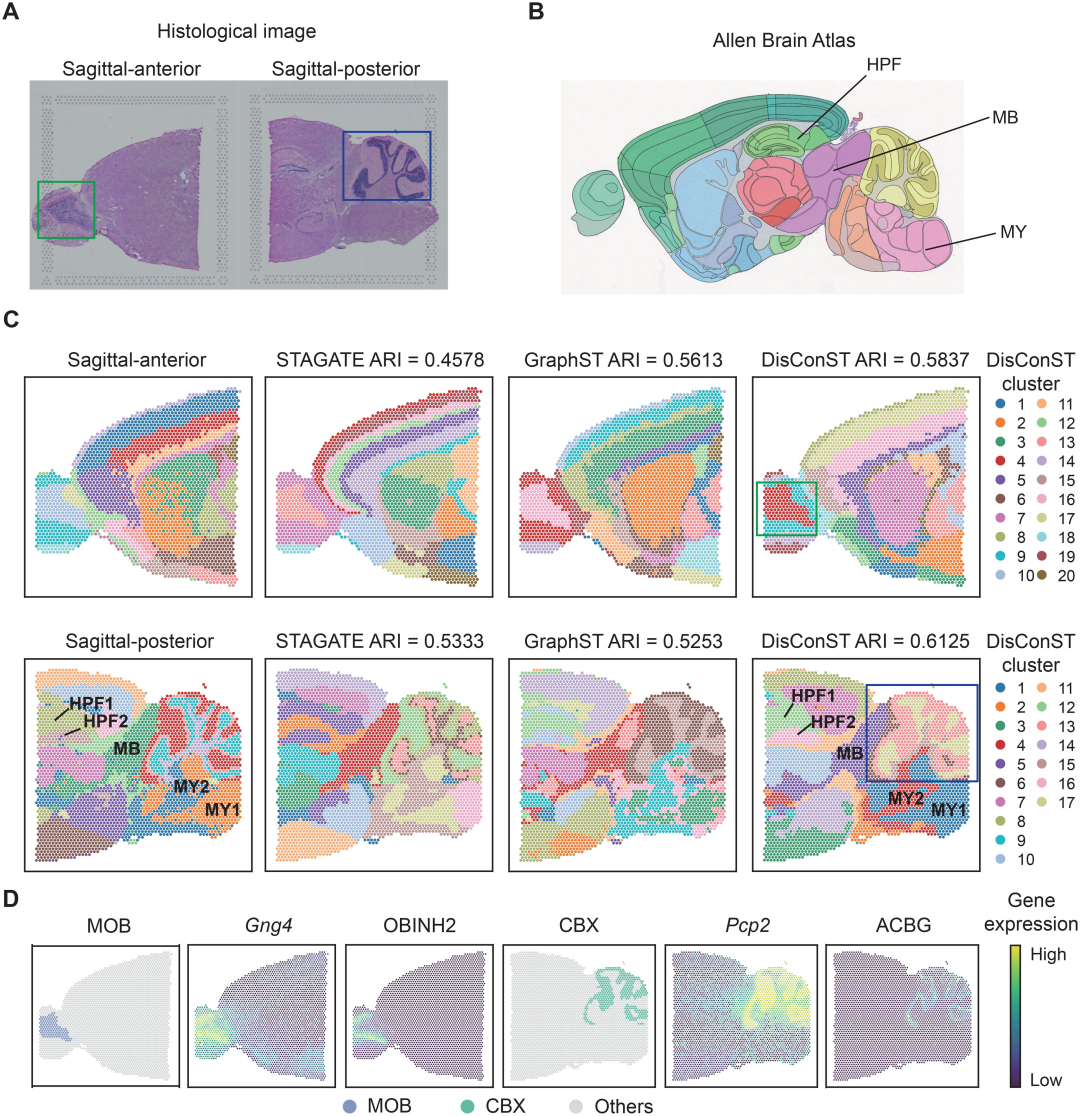
DisConST effectively identifies tissue structure in the mouse brain **A**. Histological images of sagittal-anterior and sagittal-posterior sections of the mouse brain. **B**. The Allen Brain Atlas for sagittal mouse brain at position 121. Image credit: Allen Institute for Brain Science (Allen Reference Atlas – Mouse Brain; available at https://atlas.brain-map.org). **C**. Visualization of spatial domain identification by STAGATE, GraphST, and DisConST in the sagittal-anterior and sagittal-posterior sections, alongside corresponding ground truth. **D**. Marker genes and highly prevalent cell types in the MOB and CBX. CBX, cerebellar cortex; MB, midbrain; MY, medulla; HPF, hippocampus; OBINH2, olfactory inhibitory neuron 2; ACBG, Bergmann glia of the cerebellum.

To demonstrate DisConST’s ability to identify complex organizational structures, we analyzed two mouse brain slices using 10x Visium technology. As shown in **Figure 4**A and B and Figure S6A and B, each slice is divided into anterior and posterior sagittal portions, forming the complete brain structure [48]. In Figure 4C and Figure S6C, we visualized the ground truth domain distribution from Spatial TranscriptOmics DataBase (STOmicsDB) [49], alongside spatial domain identification results from three methods applied to the two mouse brain slices. DisConST consistently outperforms STAGATE and GraphST in terms of ARI scores across both slices. Notably, DisConST accurately identifies the small olfactory bulb and the complex cerebellar cortex tissues (highlighted with green and blue boxes, respectively). Moreover, the domains corresponding to the midbrain (MB), medulla (MY), and hippocampus (HPF) regions, marked as MB, MY1/2, and HPF1/2 in Figure 4C, align well with the ground truth. The comparison with stLearn, SEDR, SpaGCN, CCST, and BayesSpace is shown in Figure S5.

Furthermore, the spatial expression patterns of marker genes and the distribution of corresponding cell types further validate the accuracy of the tissue identification. As shown in Figure 4D and Figure S6D, the protein-coding gene *Gng4* exhibits high expression in the MOB tissue [50]. Similarly, olfactory inhibitory neuron 2 (OBINH2), a kind of olfactory inhibitory neurons, is also significantly concentrated in the same region [51]. These observations align well with the MOB region identified by DisConST. Additionally, *Pcp2*, a gene encoding Purkinje cell protein 2 [52], is highly expressed in Purkinje cells of the mammalian cerebellum, while Bergmann glia of the cerebellum (ACBG) [51] is distinctly found in the cerebellum. This supports DisConST’s accurate identification of the cerebellar region. The alignment of marker gene expression and cell-type distribution with DisConST’s results further confirms the precision of its tissue structure identification.

To further demonstrate the accuracy of DisConST in tissue structure identification, we assembled the anterior and posterior sagittal regions into a unified and complete brain tissue slice to identify the coherent spatial domains simultaneously across the junction. As shown in Figure S7, DisConST not only successfully detects contiguous and coherent spatial domains precisely at the slice junctions, but also exhibits a remarkable ability to precisely identify several other vital brain tissues. The Purkinje cell layer in the cerebellum and two HPF regions were precisely pinpointed on the whole slice. These findings affirm the proficiency of DisConST in handling complex tissue architectures and accurately identifying spatial domains even in assembled brain tissue slices.

### DisConST accurately identifies the tissue structures of mouse embryo

**Figure 5:**
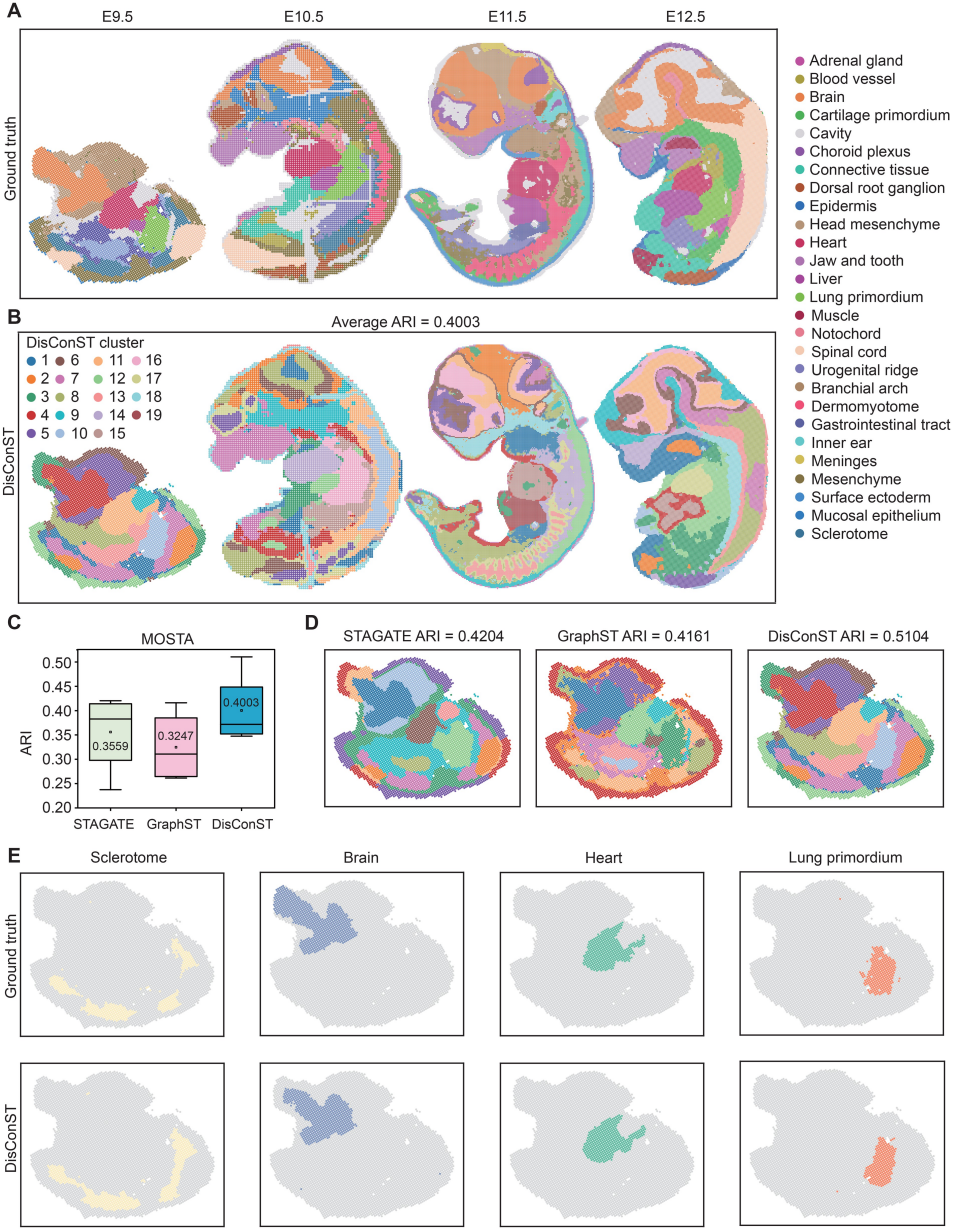
DisConST accurately identifies tissue structures in mouse embryos **A**. Ground truth of the MOSTA at four different developmental stages (E9.5–E12.5). **B**. Spatial domains detected by DisConST across the four MOSTA datasets. **C**. Box plot of ARI scores for spatial domains identified by STAGATE, GraphST, and DisConST on the MOSTA datasets. The center line, box limits, and whiskers represent the median, upper and lower quartiles, and 1.5× interquartile range, respectively. The average ARI score is also indicated within the box plot. **D**. Visualization of spatial domains detected by the three methods on the MOSTA E9.5 dataset. **E**. Spatial domains detected by DisConST with corresponding annotations for four tissues or organs: sclerotome, brain, heart, and lung primordium. E9.5, embryonic day 9.5.

Research on embryonic development is a critical application of ST technology. By analyzing the spatial distribution of transcripts within embryos, we can gain valuable insights into tissue and organ formation and differentiation, providing a deeper understanding of these processes from a spatial perspective. Specifically, ST at various developmental stages enables us to observe the dynamic progression of embryonic development. To evaluate DisConST’s effectiveness in identifying organ and tissue structures in developing embryos, we applied it to the MOSTA data from four developmental stages, including E9.5, E10.5, E11.5, and E12.5, generated using Stereo-seq technology [40]. As illustrated in **Figure 5**A, manual annotations capture the formation and developmental processes of multiple organs. As shown in Figure 5B, DisConST accurately identifies the overall structures of embryonic tissues and organs across all four slices, achieving an average ARI of 0.4003, which outperforms STAGATE and GraphST, average ARI scores are 0.3559 and 0.3247, respectively (Figure 5C).

Using stage E9.5 as an example, Figure 5D highlights DisConST’s superior performance, achieving an ARI score of 0.5104, approximately 25% higher than STAGATE and GraphST. DisConST successfully identifies domains with well- defined boundaries and organ shapes that closely align with the ground truth, including the sclerotome, brain, heart, and lung primordium, as shown in Figure 5E. Furthermore, as shown in Figure S8, the marker genes specific to these organs further evaluated the spatial domain identification of DisConST. In contrast, GraphST produces blurred domain boundaries, and STAGATE inaccurately captures the shapes of key organs, such as the heart. For replication purposes, the performance was also evaluated in the other four slices at stage E9.5, as shown in Figure S9.

The comparison with SEDR, SpaGCN, CCST, GraphST, and STAGATE at all four stages is shown in Figure S10. A comparative analysis of spatial domain identification across the four stages of embryonic development further demonstrates the superior performance of DisConST in cross-stage identification of organizational structures. As illustrated in Figure S11, DisConST consistently identifies the heart structure in all four stages. Additionally, despite changes in the jaw and tooth structures observed in the last three stages, DisConST still achieves accurate identification. Moreover, DisConST detects the appearance and structure of muscle tissue, which emerges at the E12.5 stage. In summary, DisConST effectively reveals the early embryonic process of organ formation and tissue structuring, offering valuable insights for embryonic development research.

### DisConST effectively dissects the tumor immune microenvironment

ST plays a crucial role in tumor research by preserving the spatial distribution of tumor domains, thereby providing valuable insights into the tumor microenvironment. To evaluate the effectiveness of DisConST in analyzing the immune microenvironment, we applied it to ST data from breast cancer using 10x Visium technology. This dataset exhibits high heterogeneity and complex domain structures [53], contrasting with the distinct hierarchical organization and high similarity between layers observed in the DLPFC dataset.

**Figure 6:**
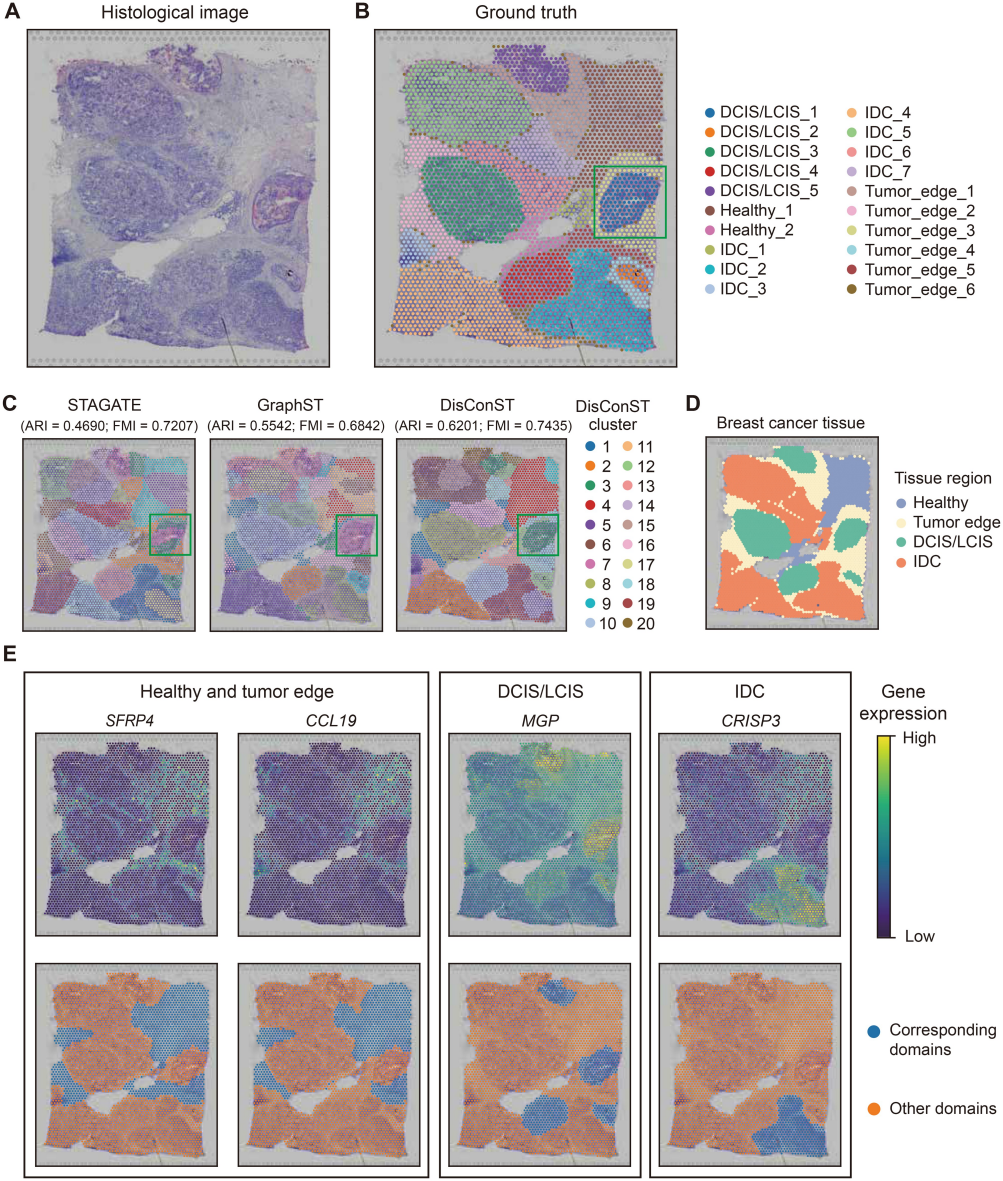
DisConST effectively dissects the immune microenvironment **A**. Histological image of a human breast cancer slice. **B**. Ground truth of the breast cancer tissue. **C**. Spatial domains identified by STAGATE, GraphST, and DisConST in the breast cancer tissue. **D**. Healthy, tumor edge, DCIS/LCIS, and IDC regions of the breast cancer tissue, annotated according to the ground truth. **E**. Genes highly expressed in healthy and tumor edge regions, DCIS/LCIS regions, and IDC regions. DCIS, Ductal Carcinoma *In Situ*; LCIS, Lobular Carcinoma *In Situ*; IDC, Invasive Ductal Carcinoma; FMI, Fowlkes–Mallows Index.

As illustrated in **Figure 6**A and B, the histological image is divided into 20 domains based on pathological features. To highlight the accuracy of DisConST, we compared its spatial domain identification results with those of STAGATE and GraphST. Considering that a tumor region may consist of multiple subclones, relying only on the ARI metric for evaluation might overlook this inherent characteristic of tumor data. Therefore, we employed both ARI and FMI to comprehensively evaluate the performance. Prior to FMI calculation, we performed label transformation to align predicted labels with ground truth labels. As illustrated in Figure 6C, DisConST demonstrates superior performance over both comparative methods across these two metrics. Specifically, DisConST not only accurately identifies the Ductal Carcinoma *In Situ*/Lobular Carcinoma *In Situ*_1 (DCIS/LCIS_1) region marked by the green box but also successfully delineates the outer tumor edge area, whereas neither GraphST nor STAGATE is able to achieve this. These results demonstrate that our spatial domain identification results preserve more detailed information. Furthermore, as shown in Figure S12, after label conversion, DisConST preserves more categories in comparison to STAGATE and GraphST, suggesting that our segmentation of each domain is more refined. These results demonstrate that DisConST can accurately delineate the distribution of tumor domains. The comparison with stLearn, SEDR, SpaGCN, CCST, and BayesSpace is shown in Figure S13.

To further demonstrate DisConST’s ability to distinguish between diseased and healthy areas, we validated our spatial domain identification results using the spatial expression patterns of marker genes. As shown in Figure 6D, this slice was divided into four regions based on manual annotation: the DCIS/LCIS area, the Invasive Ductal Carcinoma (IDC) area, the tumor edge, and the healthy area. Corresponding to these areas, we selected four marker genes — *SFRP4* [54], *CCL19* [55], *MGP* [56], and *CRISP3* [57] — which are crucial in the development or resistance of breast cancer. *SFRP4* and *CCL19* are notably expressed at high levels in the healthy area and tumor edge, while *MGP* and *CRISP3* demonstrate elevated expression in the DCIS/LCIS and IDC regions, respectively. As illustrated in Figure 6E, the expression patterns of *SFRP4* and *CCL19* align well with DisConST’s identification of domains corresponding to the healthy and tumor edge regions. The expression pattern of *MGP* correlates with domains 3, 10, and 12, which correspond to DCIS/LCIS in the ground truth. Similarly, *CRISP3* is associated with domains 14 and 19, corresponding to IDC in the ground truth. These findings highlight DisConST’s capability to accurately identify the distribution of both tumor and healthy regions.

On another breast cancer slice, we conducted a similar study. Figure S14A shows the histological image. As shown in Figure S14B, we used the Louvain algorithm to divide the slice into 17 domains. We selected nine domains that may be cancer regions. The positions of these nine domains are shown in Figure S14C, which are consistent with the staining of the histological image. In addition, as shown in Figure S14D, the marker genes of these nine domains, *DHCR24* [58], *FOXA1* [59], *MUCL1* [60], *TPD52* [61], *APOC1* [62], *FGB* [63], *TMC5* [64], and *SPP1* [65], which have been verified to have connections with the proliferation, invasion, and other processes of breast cancer, provide evidence for the identification of these nine cancer regions. In conclusion, DisConST’s precise delineation of tumor structures and its effective differentiation between healthy and tumor areas confirm its robustness in analyzing the immune microenvironment.

## Discussion and conclusion

ST has revolutionized the study of GE by offering unprecedented insights into the spatial organization of tissues. By providing spatially resolved GE data at the spot or cellular level, it enables researchers to explore tissue structure, biological development, and disease mechanisms. A critical task within ST is spatial domain identification — defining regions with similar GE patterns, which is essential for understanding tissue organization and functionality.

Despite the advances in ST, current methods for spatial domain identification often face challenges, particularly in integrating spatial and transcriptomic data, as well as in handling high dropout rates in GE profiles. To address these limitations, we introduce DisConST, a novel deep learning method specifically designed to improve spatial domain detection. DisConST integrates spatial information, transcriptomic data, and CTPs within spots, utilizing advanced optimization techniques including ZINB distribution fitting and GCL. As shown in Figure S15, these innovations allow DisConST to extract more informative latent representations, leading to superior accuracy in spatial domain recognition.

Through extensive validation on diverse datasets — ranging from tissues, organs, and embryos across various sequencing platforms, under both normal and diseased conditions — DisConST demonstrates consistently higher accuracy than existing state-of-the-art methods. Its robust performance highlights its ability to address key challenges in ST research. Moreover, the practical utility of DisConST extends to a range of biological contexts, including advancing our understanding of tissue organization, embryonic development, and the tumor immune microenvironment.

In conclusion, DisConST represents a significant leap forward in the field of ST, offering a powerful tool for accurate spatial domain identification through its innovative, distribution-aware contrastive learning approach. By addressing key limitations of existing methods, DisConST has the potential to enhance both fundamental biological research and translational applications in disease studies.

## Code availability

Source code and tutorials of DisConST, as well as the embeddings generated for each dataset, are available on GitHub (https://github.com/Zhenpm/DisConST/). The source code and embeddings generated for each dataset have also been submitted to BioCode at the National Genomics Data Center (NGDC), China National Center for Bioinformation (CNCB) (BioCode: BT007854), which are publicly accessible at https://ngdc.cncb.ac.cn/biocode/tool/BT007854.

## Supplementary Material

qzaf085_Supplementary_Data

## CRediT author statement

**Peimeng Zhen:** Methodology, Software, Visualization, Writing – original draft, Writing – review & editing. **Xiaofei Wang:** Conceptualization, Methodology, Software. **Han Shu:** Investigation, Software, Visualization. **Jialu Hu:** Formal analysis, Validation. **Yongtian Wang:** Formal analysis, Validation. **Jiajie Peng:** Formal analysis, Validation. **Xuequn Shang:** Project administration, Supervision. **Jing Chen:** Conceptualization, Writing – original draft. **Tao Wang:** Conceptualization, Funding acquisition, Resources, Writing – original draft, Writing – review & editing. All authors have read and approved the final manuscript.

## References

[1] Moses L , Pachter L. Museum of spatial transcriptomics. Nat Methods 2022;19:534–46. 35273392 10.1038/s41592-022-01409-2

[2] Wang T , Mai D, Shu H, Hu J, Wang Y, Peng J, et al Enhancing cell subpopulation discovery in cancer by integrating single-cell transcriptome and expressed variants. Fundam Res 2025;6:88–98. 41647537 10.1016/j.fmre.2025.01.001 PMC12869734

[3] Wang T , Zhao H, Xu Y, Wang Y, Shang X, Peng J, et al scMultiGAN: cell-specific imputation for single-cell transcriptomes with multiple deep generative adversarial networks. Brief Bioinform 2023;24:bbad384. 37903416 10.1093/bib/bbad384 PMC11020228

[4] Guo H , Zhang L, Cui X, Cheng L, Zhao T, Wang Y. SCancerRNA: expression at the single-cell level and interaction resource of non-coding RNA biomarkers for cancers. Genomics Proteomics Bioinformatics 2024;22:qzae023. 39341795 10.1093/gpbjnl/qzae023 PMC12016560

[5] Cao C , Wang C, Dai Q, Zou Q, Wang T. CRBPSA: circRNA-RBP interaction sites identification using sequence structural attention model. BMC Biol 2024;22:260. 39543602 10.1186/s12915-024-02055-0 PMC11566611

[6] Burgess DJ. Spatial transcriptomics coming of age. Nat Rev Genet 2019;20:317. 30980030 10.1038/s41576-019-0129-z

[7] Yang C , Liu Y, Wang X, Jia Q, Fan Y, Lu Z, et al stSNV: a comprehensive resource of SNVs in spatial transcriptome. Nucleic Acids Res 2025;53:D1224–34. 39470702 10.1093/nar/gkae945 PMC11701523

[8] Wang T , Shu H, Hu J, Wang Y, Chen J, Peng J, et al Accurately deciphering spatial domains for spatially resolved transcriptomics with stCluster. Brief Bioinform 2024;25:bbae329. 38975895 10.1093/bib/bbae329 PMC11771244

[9] Ji AL , Rubin AJ, Thrane K, Jiang S, Reynolds DL, Meyers RM, et al Multimodal analysis of composition and spatial architecture in human squamous cell carcinoma. Cell 2020;182:497–514.e22. 32579974 10.1016/j.cell.2020.05.039 PMC7391009

[10] Crosetto N , Bienko M, van Oudenaarden A. Spatially resolved transcriptomics and beyond. Nat Rev Genet 2015;16:57–66. 25446315 10.1038/nrg3832

[11] Moor AE , Itzkovitz S. Spatial transcriptomics: paving the way for tissue-level systems biology. Curr Opin Biotechnol 2017;46:126–33. 28346891 10.1016/j.copbio.2017.02.004

[12] Pham D , Tan X, Balderson B, Xu J, Grice LF, Yoon S, et al Robust mapping of spatiotemporal trajectories and cell–cell interactions in healthy and diseased tissues. Nat Commun 2023;14:7739. 38007580 10.1038/s41467-023-43120-6 PMC10676408

[13] Xu H , Fu H, Long Y, Ang KS, Sethi R, Chong K, et al Unsupervised spatially embedded deep representation of spatial transcriptomics. Genome Med 2024;16:12. 38217035 10.1186/s13073-024-01283-x PMC10790257

[14] Hu J , Li X, Coleman K, Schroeder A, Ma N, Irwin DJ, et al SpaGCN: integrating gene expression, spatial location and histology to identify spatial domains and spatially variable genes by graph convolutional network. Nat Methods 2021;18:1342–51. 34711970 10.1038/s41592-021-01255-8

[15] Li J , Chen S, Pan X, Yuan Y, Shen HB. Cell clustering for spatial transcriptomics data with graph neural networks. Nat Comput Sci 2022;2:399–408. 38177586 10.1038/s43588-022-00266-5

[16] Zhao E , Stone MR, Ren X, Guenthoer J, Smythe KS, Pulliam T, et al Spatial transcriptomics at subspot resolution with BayesSpace. Nat Biotechnol 2021;39:1375–84. 34083791 10.1038/s41587-021-00935-2 PMC8763026

[17] Dong K , Zhang S. Deciphering spatial domains from spatially resolved transcriptomics with an adaptive graph attention auto-encoder. Nat Commun 2022;13:1739. 35365632 10.1038/s41467-022-29439-6 PMC8976049

[18] Long Y , Ang KS, Li M, Chong KLK, Sethi R, Zhong C, et al Spatially informed clustering, integration, and deconvolution of spatial transcriptomics with GraphST. Nat Commun 2023;14:1155. 36859400 10.1038/s41467-023-36796-3 PMC9977836

[19] Zhu P , Shu H, Wang Y, Wang X, Zhao Y, Hu J, et al MAEST: accurately spatial domain detection in spatial transcriptomics with graph masked autoencoder. Brief Bioinform 2025;26:bbaf086. 40052440 10.1093/bib/bbaf086 PMC11886571

[20] Wang J , Xiao Y, Shang X, Peng J. Predicting drug–target binding affinity with cross-scale graph contrastive learning. Brief Bioinform 2023;25:bbad516. 38221904 10.1093/bib/bbad516 PMC10788681

[21] Yau KKW , Wang K, Lee AH. Zero-inflated negative binomial mixed regression modeling of over-dispersed count data with extra zeros. Biom J 2003;45:437–52.

[22] Tian T , Wan J, Song Q, Wei Z. Clustering single-cell RNA-seq data with a model-based deep learning approach. Nat Mach Intell 2019;1:191–8.

[23] Wolf FA , Angerer P, Theis FJ. SCANPY: large-scale single-cell gene expression data analysis. Genome Biol 2018;19:15. 29409532 10.1186/s13059-017-1382-0 PMC5802054

[24] Xu H , Wang S, Fang M, Luo S, Chen C, Wan S, et al SPACEL: deep learning-based characterization of spatial transcriptome architectures. Nat Commun 2023;14:7603. 37990022 10.1038/s41467-023-43220-3 PMC10663563

[25] Biancalani T , Scalia G, Buffoni L, Avasthi R, Lu Z, Sanger A, et al Deep learning and alignment of spatially resolved single-cell transcriptomes with Tangram. Nat Methods 2021;18:1352–62. 34711971 10.1038/s41592-021-01264-7 PMC8566243

[26] Veličković P , Cucurull G, Casanova A, Romero A, Liò P, Bengio Y. Graph attention networks. The 6th International Conference on Learning Representations 2018.

[27] Zhang T , Zhang X, Wu Z, Ren J, Zhao Z, Zhang H, et al VGAE-CCI: variational graph autoencoder-based construction of 3D spatial cell–cell communication network. Brief Bioinform 2025;26:bbae619. 10.1093/bib/bbae619 PMC11586124 39581873

[28] Scrucca L , Fop M, Murphy TB, Raftery AE. mclust 5: clustering, classification and density estimation using Gaussian finite mixture models. R J 2016;8:289–317. 27818791 PMC5096736

[29] De Meo P , Ferrara E, Fiumara G, Provetti A. Generalized louvain method for community detection in large networks. 11th International Conference on Intelligent Systems Design and Applications 2011:88–93.

[30] Yeung KY , Ruzzo WL. Principal component analysis for clustering gene expression data. Bioinformatics 2001;17:763–74. 11590094 10.1093/bioinformatics/17.9.763

[31] Romano S , Bailey J, Nguyen V, Verspoor K. Standardized mutual information for clustering comparisons: one step further in adjustment for chance. Proceedings of the 31st International Conference on Machine Learning 2014;32:1143–51.

[32] Knops ZF , Maintz JB, Viergever MA, Pluim JP. Normalized mutual information based registration using k-means clustering and shading correction. Med Image Anal 2006;10:432–9. 16111913 10.1016/j.media.2005.03.009

[33] Chicco D , Jurman G. A statistical comparison between Matthews correlation coefficient (MCC), prevalence threshold, and Fowlkes*-*Mallows index. J Biomed Inform 2023;144:104426. 37352899 10.1016/j.jbi.2023.104426

[34] Wickham H. ggplot2. Wiley Interdiscip Rev Comput Stat 2011;3:180–5.

[35] Becht E , McInnes L, Healy J, Dutertre CA, Kwok IWH, Ng LG, et al Dimensionality reduction for visualizing single-cell data using UMAP. Nat Biotechnol 2019;37:38–44. 10.1038/nbt.4314 30531897

[36] Wolf FA , Hamey FK, Plass M, Solana J, Dahlin JS, Göttgens B, et al PAGA: graph abstraction reconciles clustering with trajectory inference through a topology preserving map of single cells. Genome Biol 2019;20:59. 30890159 10.1186/s13059-019-1663-x PMC6425583

[37] Maynard KR , Collado-Torres L, Weber LM, Uytingco C, Barry BK, Williams SR, et al Transcriptome-scale spatial gene expression in the human dorsolateral prefrontal cortex. Nat Neurosci 2021;24:425–36. 33558695 10.1038/s41593-020-00787-0 PMC8095368

[38] Wei X , Fu S, Li H, Liu Y, Wang S, Feng W, et al Single-cell Stereo-seq reveals induced progenitor cells involved in axolotl brain regeneration. Science 2022;377:eabp9444. 36048929 10.1126/science.abp9444

[39] Ståhl PL , Salmén F, Vickovic S, Lundmark A, Navarro JF, Magnusson J, et al Visualization and analysis of gene expression in tissue sections by spatial transcriptomics. Science 2016;353:78–82. 27365449 10.1126/science.aaf2403

[40] Chen A , Liao S, Cheng M, Ma K, Wu L, Lai Y, et al Spatiotemporal transcriptomic atlas of mouse organogenesis using DNA nanoball-patterned arrays. Cell 2022;185:1777–92.e21. 35512705 10.1016/j.cell.2022.04.003

[41] Lohoff T , Ghazanfar S, Missarova A, Koulena N, Pierson N, Griffiths JA, et al Integration of spatial and single-cell transcriptomic data elucidates mouse organogenesis. Nat Biotechnol 2022;40:74–85. 34489600 10.1038/s41587-021-01006-2 PMC8763645

[42] Li H , Ruberu K, Karl T, Garner B. Cerebral apolipoprotein-D is hypoglycosylated compared to peripheral tissues and is variably expressed in mouse and human brain regions. PLoS One 2016;11:e0148238. 26829325 10.1371/journal.pone.0148238 PMC4734669

[43] Ma J , Dankulich-Nagrudny L, Lowe G. Cholecystokinin: an excitatory modulator of mitral/tufted cells in the mouse olfactory bulb. PLoS One 2013;8:e64170. 23691163 10.1371/journal.pone.0064170 PMC3655022

[44] Sun S , Zhu J, Zhou X. Statistical analysis of spatial expression patterns for spatially resolved transcriptomic studies. Nat Methods 2020;17:193–200. 31988518 10.1038/s41592-019-0701-7 PMC7233129

[45] Lin Y , Brosius J, Tiedge H. Neuronal BC1 RNA: co-expression with growth-associated protein-43 messenger RNA. Neuroscience 2001;103:465–79. 11246161 10.1016/s0306-4522(01)00003-3

[46] Perera SN , Williams RM, Lyne R, Stubbs O, Buehler DP, Sauka-Spengler T, et al Insights into olfactory ensheathing cell development from a laser-microdissection and transcriptome-profiling approach. Glia 2020;68:2550–84. 32857879 10.1002/glia.23870 PMC7116175

[47] Uchigashima M , Cheung A, Suh J, Watanabe M, Futai K. Differential expression of neurexin genes in the mouse brain. J Comp Neurol 2019;527:1940–65. 30761534 10.1002/cne.24664 PMC6592846

[48] Wang Q, , Ding SL, , Li Y, Royall J, Feng D, Lesnar P, et al. The Allen Mouse Brain Common Coordinate Framework: a 3D reference atlas. Cell 2020;181:936–53.e20. 32386544 10.1016/j.cell.2020.04.007 PMC8152789

[49] Xu Z , Wang W, Yang T, Li L, Ma X, Chen J, et al STOmicsDB: a comprehensive database for spatial transcriptomics data sharing, analysis and visualization. Nucleic Acids Res 2024;52:D1053–61. 37953328 10.1093/nar/gkad933 PMC10767841

[50] Zeng HL , Rao X, Zhang LK, Zhao X, Zhang WP, Wang J, et al Quantitative proteomics reveals olfactory input-dependent alterations in the mouse olfactory bulb proteome. J Proteomics 2014;109:125–42. 24998433 10.1016/j.jprot.2014.06.023

[51] Zeisel A , Hochgerner H, Lönnerberg P, Johnsson A, Memic F, van der Zwan J, et al Molecular architecture of the mouse nervous system. Cell 2018;174:999–1014.e22. 30096314 10.1016/j.cell.2018.06.021 PMC6086934

[52] Vandaele S , Nordquist DT, Feddersen RM, Tretjakoff I, Peterson AC, Orr HT. Purkinje cell protein-2 regulatory regions and transgene expression in cerebellar compartments. Genes Dev 1991;5:1136–48. 2065970 10.1101/gad.5.7.1136

[53] Polyak K. Heterogeneity in breast cancer. J Clin Invest 2011;121:3786–8. 21965334 10.1172/JCI60534 PMC3195489

[54] Zhang L , Liu C, Li Z. Research progress of secreted frizzled-related proteins in female malignant tumor. Cancer Res Prev Treat 2020;47:218–22.

[55] Wang J , Qin D, Ye L, Wan L, Wang F, Yang Y, et al CCL19 has potential to be a potential prognostic biomarker and a modulator of tumor immune microenvironment (TIME) of breast cancer: a comprehensive analysis based on TCGA database. Aging (Albany NY) 2022;14:4158–75. 35550569 10.18632/aging.204081 PMC9134962

[56] Yoshimura K , Takeuchi K, Nagasaki K, Ogishima S, Tanaka H, Iwase T, et al Prognostic value of matrix Gla protein in breast cancer. Mol Med Rep 2009;2:549–53. 21475864 10.3892/mmr_00000135

[57] Wang Y , Sheng N, Xie Y, Chen S, Lu J, Zhang Z, et al Low expression of CRISP3 predicts a favorable prognosis in patients with mammary carcinoma. J Cell Physiol 2019;234:13629–38. 30609035 10.1002/jcp.28043 PMC13483335

[58] Qiu T , Cao J, Chen W, Wang J, Wang Y, Zhao L, et al 24-Dehydrocholesterol reductase promotes the growth of breast cancer stem-like cells through the Hedgehog pathway. Cancer Sci 2020;111:3653–64. 32713162 10.1111/cas.14587 PMC7540995

[59] Nakshatri H , Badve S. FOXA1 in breast cancer. Expert Rev Mol Med 2009;11:e8. 19261198 10.1017/S1462399409001008

[60] Miksicek RJ , Myal Y, Watson PH, Walker C, Murphy LC, Leygue E. Identification of a novel breast- and salivary gland-specific, mucin-like gene strongly expressed in normal and tumor human mammary epithelium. Cancer Res 2002;62:2736–40. 12019145

[61] Wang Y , Fang J, Gu F. miR-125b-5p/TPD52 axis affects proliferation, migration and invasion of breast cancer cells. Mol Biotechnol 2022;64:1003–12. 35320453 10.1007/s12033-022-00475-3

[62] Zhang H , Wang Y, Liu C, Li W, Zhou F, Wang X, et al The apolipoprotein C1 is involved in breast cancer progression via EMT and MAPK/JNK pathway. Pathol Res Pract 2022;229:153746. 34952429 10.1016/j.prp.2021.153746

[63] Liu H , Xiang L, Mei Y. miR-877-5p inhibits epithelial mesenchymal transformation of breast cancer cells by targeting FGB. Dis Markers 2022;2022:4882375. 36438895 10.1155/2022/4882375 PMC9691316

[64] Zhang H , Zhang X, Xu W, Wang J. TMC5 is highly expressed in human cancers and corelates to prognosis and immune cell infiltration: a comprehensive bioinformatics analysis. Front Mol Biosci 2022;8:810864. 35096973 10.3389/fmolb.2021.810864 PMC8792843

[65] Göthlin Eremo A , Lagergren K, Othman L, Montgomery S, Andersson G, Tina E. Evaluation of *SPP1/osteopontin* expression as predictor of recurrence in tamoxifen treated breast cancer. Sci Rep 2020;10:1451. 31996744 10.1038/s41598-020-58323-w PMC6989629

