# Supplementary material for: DisConST: Distribution-aware Contrastive Learning for Spatial Domain Identification
Source: Genomics Proteomics Bioinformatics. 2025 Sep 24;24(1):qzaf085. doi: 10.1093/gpbjnl/qzaf085 (PMC13317986; doi:10.1093/gpbjnl/qzaf085)
Supplement: qzaf085_Supplementary_Data [file qzaf085_supplementary_data.zip › Figure S11 (1).pdf]

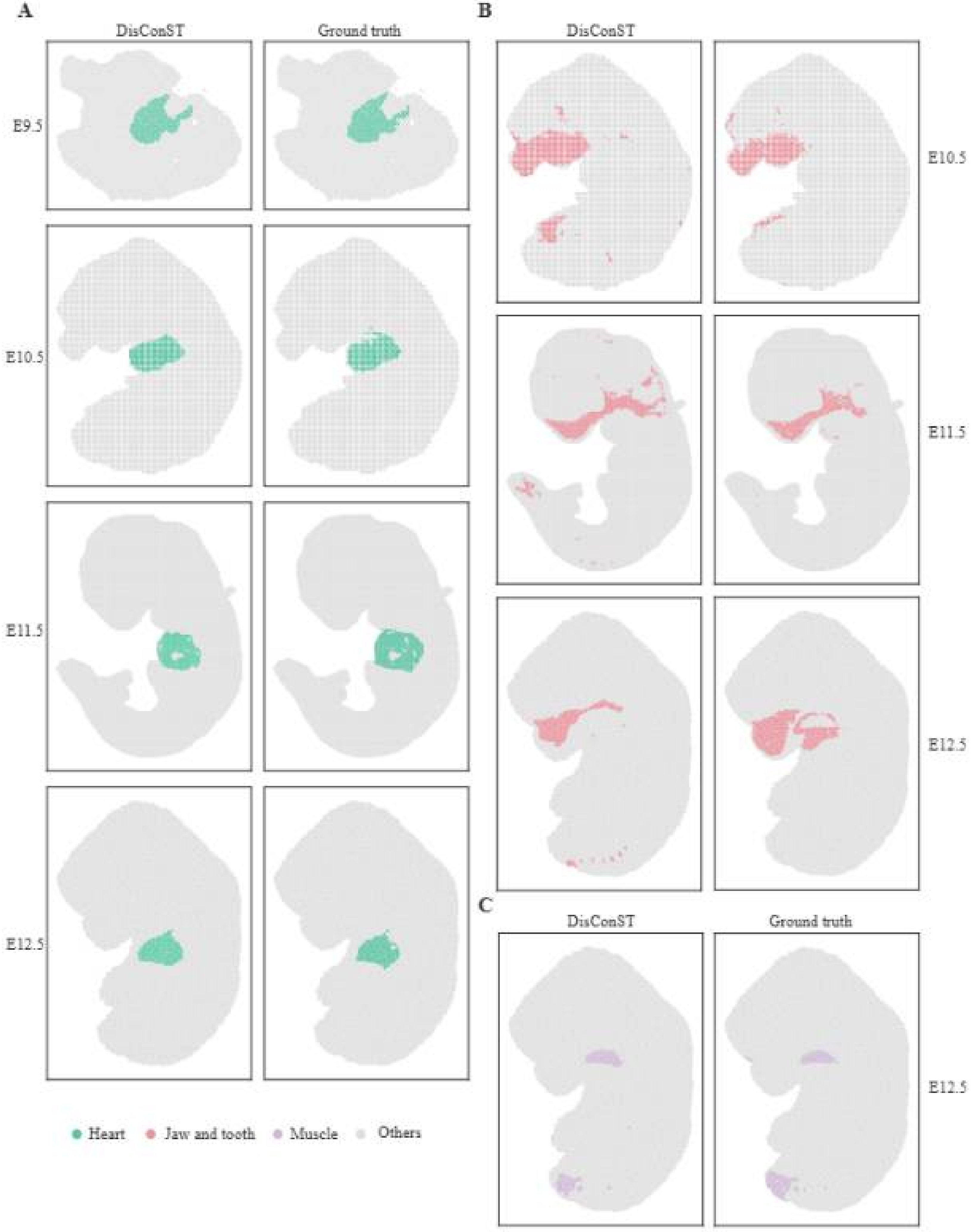
A
DisConST
Ground truth
E9.5
E10.5
E11.5
E12.5
B
DisConST
E10.5
E11.5
E12.5
C
DisConST
Ground truth
E12.5
Heart
Jaw and tooth
Muscle
Others
