# Supplementary material for: DisConST: Distribution-aware Contrastive Learning for Spatial Domain Identification
Source: Genomics Proteomics Bioinformatics. 2025 Sep 24;24(1):qzaf085. doi: 10.1093/gpbjnl/qzaf085 (PMC13317986; doi:10.1093/gpbjnl/qzaf085)
Supplement: qzaf085_Supplementary_Data [file qzaf085_supplementary_data.zip › Figure S12 (1).pdf]

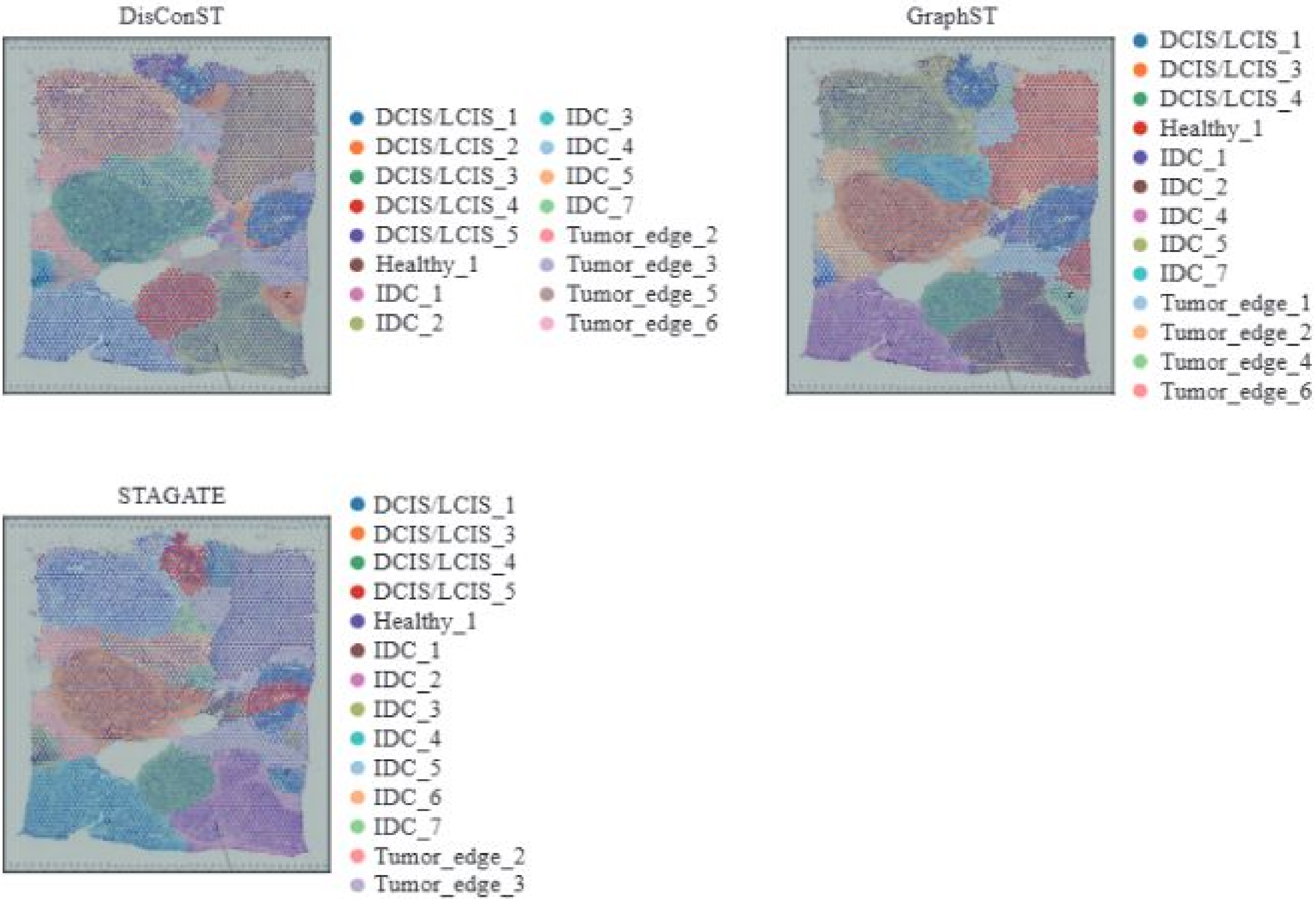
DisConST
DCIS/LCIS_1
DCIS/LCIS_2
DCIS/LCIS_3
DCIS/LCIS_4
DCIS/LCIS_5
Healthy_1
IDC_1
IDC_2
IDC_3
IDC_4
IDC_5
IDC_7
Tumor_edge_2
Tumor_edge_3
Tumor_edge_5
Tumor_edge_6
GraphST
DCIS/LCIS_1
DCIS/LCIS_3
DCIS/LCIS_4
Healthy_1
IDC_1
IDC_2
IDC_4
IDC_5
IDC_7
Tumor_edge_1
Tumor_edge_2
Tumor_edge_4
Tumor_edge_6
STAGATE
DCIS/LCIS_1
DCIS/LCIS_3
DCIS/LCIS_4
DCIS/LCIS_5
Healthy_1
IDC_1
IDC_2
IDC_3
IDC_4
IDC_5
IDC_6
IDC_7
Tumor_edge_2
Tumor_edge_3
