# Supplementary material for: DisConST: Distribution-aware Contrastive Learning for Spatial Domain Identification
Source: Genomics Proteomics Bioinformatics. 2025 Sep 24;24(1):qzaf085. doi: 10.1093/gpbjnl/qzaf085 (PMC13317986; doi:10.1093/gpbjnl/qzaf085)
Supplement: qzaf085_Supplementary_Data [file qzaf085_supplementary_data.zip › Figure S14 (1).pdf]

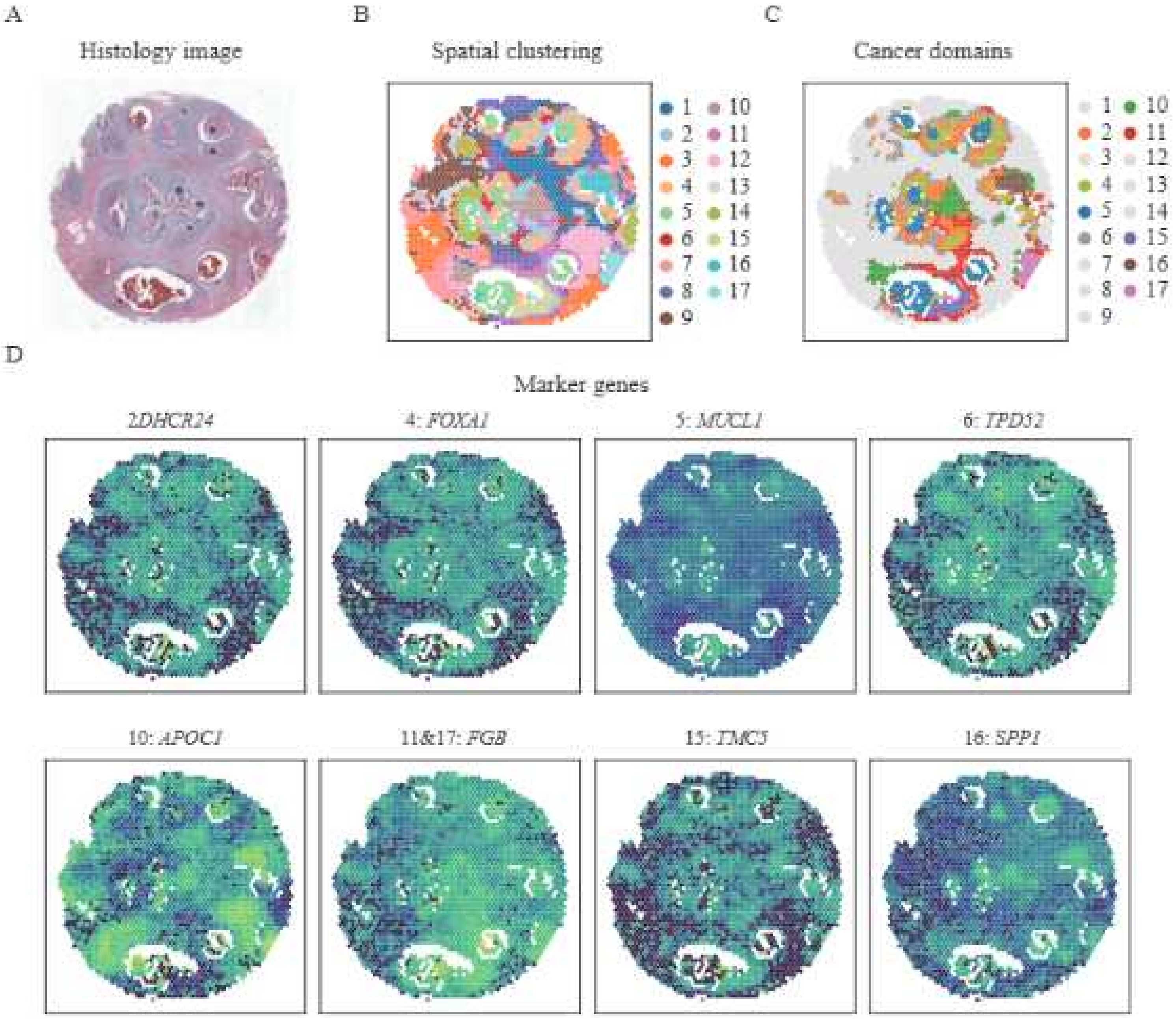

A
Histology image
B
Spatial clustering
1 2 3 4 5 6 7 8 9 10 11 12 13 14 15 16 17
C
Cancer domains
1 2 3 4 5 6 7 8 9 10 11 12 13 14 15 16 17
D
Marker genes
2DHCR24
4: FOXA1
5: MUCL1
6: TPD52
10: APOC1
11&17: FGB
15: TMC5
16: SPP1
