# Supplementary material for: DisConST: Distribution-aware Contrastive Learning for Spatial Domain Identification
Source: Genomics Proteomics Bioinformatics. 2025 Sep 24;24(1):qzaf085. doi: 10.1093/gpbjnl/qzaf085 (PMC13317986; doi:10.1093/gpbjnl/qzaf085)
Supplement: qzaf085_Supplementary_Data [file qzaf085_supplementary_data.zip › Figure S6 (1).pdf]

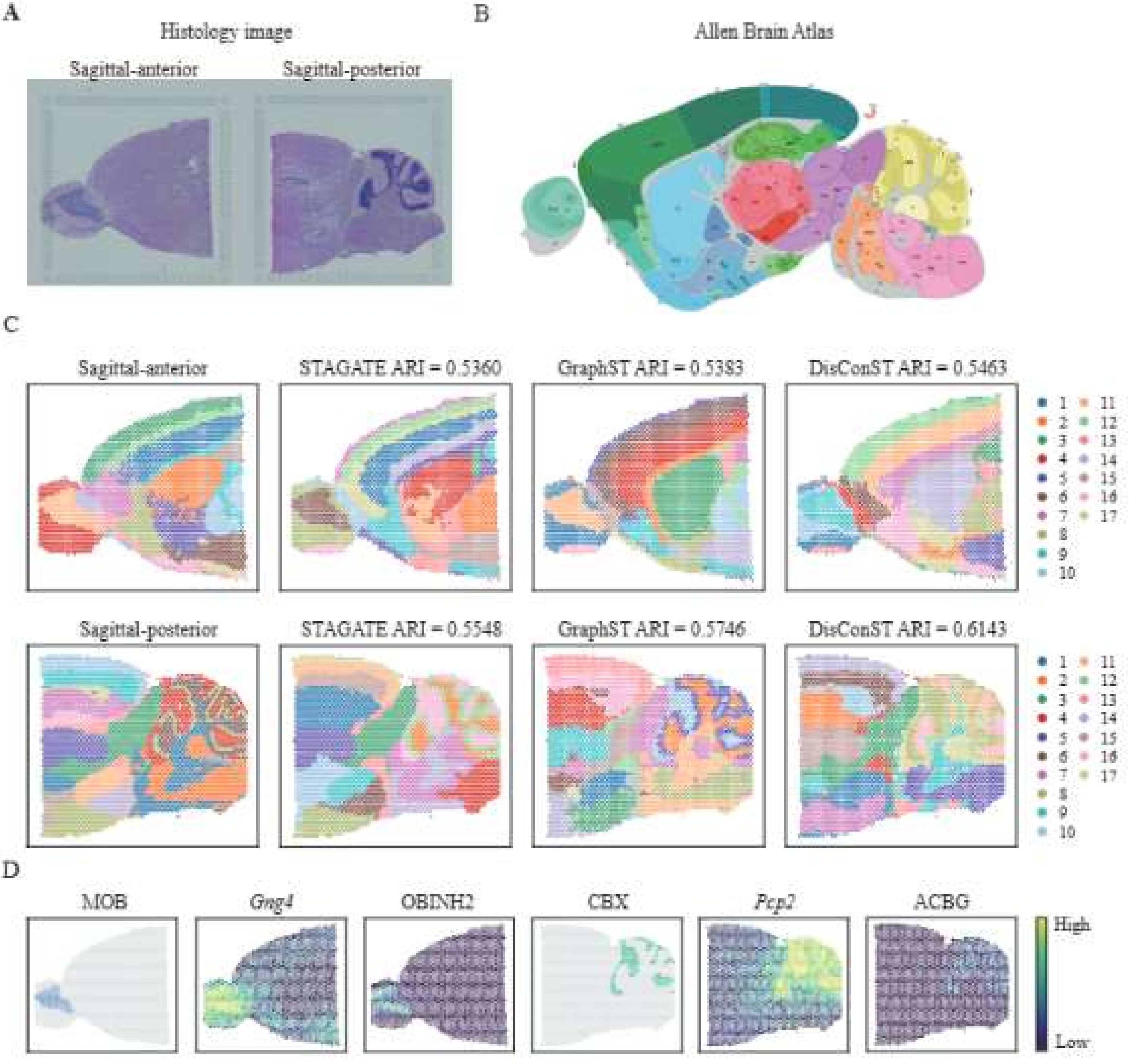

A
Histology image
Sagittal-anterior
Sagittal-posterior
B
Allen Brain Atlas
C
Sagittal-anterior
STAGATE ARI = 0.5360
GraphST ARI = 0.5383
DisConST ARI = 0.5463
1 2 3 4 5 6 7 8 9 10 11 12 13 14 15 16 17
Sagittal-posterior
STAGATE ARI = 0.5548
GraphST ARI = 0.5746
DisConST ARI = 0.6143
1 2 3 4 5 6 7 8 9 10 11 12 13 14 15 16 17
D
MOB
Gng4
OBINH2
CBX
Pcp2
ACBG
High
Low
