# Supplementary material for: DisConST: Distribution-aware Contrastive Learning for Spatial Domain Identification
Source: Genomics Proteomics Bioinformatics. 2025 Sep 24;24(1):qzaf085. doi: 10.1093/gpbjnl/qzaf085 (PMC13317986; doi:10.1093/gpbjnl/qzaf085)
Supplement: qzaf085_Supplementary_Data [file qzaf085_supplementary_data.zip › Supplementary material captions.docx]

**Supplementary material**

**Figure S1 Spatial expression of marker gene for each cortex layer in the DLPFC dataset**

**A**. Ground truth and the spatial domain identification results of DisConST on DLPFC slice #151507. **B**. Spatial expression of common marker genes in adjacent layers. **C**. Spatial expression of specific marker gene for each layer.

**Figure S2 Ground truth and spatial domain identifications of stLearn, SEDR, SpaGCN, CCST, BayesSpace, STAGATE, GraphST, and DisConST on the 12 slices of DLPFC dataset, respectively**

**Figure S3 Spatial expression of marker genes in structures detected in the mouse olfactory bulb dataset**

**A**. Laminar structure analysis of mouse olfactory bulb based on Stereo-seq sequncing data. Figures from top to down show the ground truth, clustering results of DisConST, STAGATE, GraphST, and corresponding marker genes of eight layers from the inner to the outer. Although our identification result in the last layer differ significantly from ground truth, it is consistent with the spatial expression pattern of the marker gene. **B**. Laminar structure analysis of mouse olfactory bulb based on ST sequencing data. Figures from left to right show the spatial expression of marker genes, ground truth, and clustering results of DisConST, STAGATE, GraphST.

**Figure S4 Performance of SEDR, CCST, and SpaGCN across different sequencing platforms**

The four datasets from top to bottom are from the Stereo-seq, ST, SeqFISH, and Stereo-seq platforms, respectively. “N/A”: SEDR’s memory requirements for SeqFISH mouse embryo data exceeded the hardware limit. As stLearn requires tissue morphology, BayesSpace only provides data processing methods on the 10X Visium and ST sequencing platforms, we do not test their cross platform performance.

**Figure S5 Mouse brain tissue structure identification results of stLearn, SEDR, SpaGCN, CCST, and BayesSpace**

**Figure S6 DisConST accurately distinguishes different structures in the mouse brain**

**A**. The histology image of mouse brain sagittal-anterior and sagittal-posterior sections. **B**. The Allen Brain Atlas of the sagittal mouse brain in position 121. **C**. Visualization of spatial domain identification of three methods, STAGATE, GraphST, and DisConST in mouse brain sagittal-anterior and sagittal-posterior sections and corresponding ground truth. **D**. Marker genes and highly-distributed cell types in MOB and CBX.

**Figure S7 DisConST effectively identifies coherent tissue structures on the stitched mouse brain slices**

**A**. The histology image of mouse brain sagittal-anterior and sagittal-posterior sections. The two hippocampal regions are highlighted by yellow boxes in the histological image. **B**. The Allen Brain Atlas of the sagittal mouse brain in position 121. **C**. The spatial domains identified by DisConST. Brain tissue structures, including cortex, purkinje cell layer, and hippocampal are labeled in **B** and **C**.

**Figure S8 Spatial domain identification results and corresponding marker genes’ spatial expression in sclerotome, brain, and heart**

**Figure S9 Ground truth and spatial domain identifications of STAGATE, GraphST, and DisConST on the MOSTA E9.5 data, respectively**

E1S1, E2S1, E2S2, and E2S4 means different slices at stage E9.5.

**Figure S10 Spatial domain identifications of comparison methods SEDR, SpaGCN, CCST, GraphST, and STAGATE on the 4 slices of MOSTA dataset (E9.5, E10.5, E11.5, and E12.5), respectively**

“N/A”: SEDR’s memory requirements for SeqFISH mouse embryo data exceeded the hardware limit. As stLearn requires tissue morphology, BayesSpace only provides data processing methods on the 10X Visium and ST sequencing platforms, we do not compare them here.

**Figure S11 The domains of heart, jaw, tooth, and muscle**

**A**. Ground truth and spatial domain identifications of heart among all four stages. **B**. Ground truth and spatial domain identifications of jaw and tooth among E10.5, E11.5, and E12.5. **C**. Ground truth and spatial domain identification of muscle on the E12.5 slice.

**Figure S12 The domain identification results of DisConST, GraphST, and STAGATE after label conversion**

**Figure S13 The domain identification results of stLearn, SEDR, SpaGCN, CCST, and BayesSpace on human breast cancer dataset**

**Figure S14 The ability of DisConST to analyze the immune microenvironment**

**A**. Hitological image of the breast cancer slice. **B**. Spatial domains identified by DisConST in breast cancer tissue. **C**. Cancer domains identified by DisConST. **D**. The spatial expression of marker genes corresponding to these cancer domains.

**Figure S15 The boxplot of DisConST ablation experiment on the 12 slices of DLPFC dataset**

Gene means DisConST only uses gene expression data, cell means using cell type proportion data only, both means using these two data without GCL loss. On the contrary gene/GCL, cell/GCL and both/GCL mean using these data with GCL loss. The boxplot’s small square, center line, box limits, and whiskers denote the average, median, upper and lower quartiles, and 1.5× interquartile range, respectively. These ARI scores are calculated using unrefined labels.

**Table S1 Parameter selection on different datasets**

**Table S2 The state-of-the-art methods for spatial domain identification in spatial transcriptomics**

**Table S3 Summary of all spatial transcriptomics datasets used for experiments in the work**

**Table S4 ARI scores of DisConST and seven comparison methods on 12 DLPFC slices**

**Table S5 AMI scores of DisConST and seven comparison methods on 12 DLPFC slices**

**Table S6 NMI scores of DisConST and seven comparison methods on 12 DLPFC slices**

**Table S7 ARI scores of DisConST and seven comparison methods on all datasets**
