# Supplementary material for: DisConST: Distribution-aware Contrastive Learning for Spatial Domain Identification
Source: Genomics Proteomics Bioinformatics. 2025 Sep 24;24(1):qzaf085. doi: 10.1093/gpbjnl/qzaf085 (PMC13317986; doi:10.1093/gpbjnl/qzaf085)
Supplement: qzaf085_Supplementary_Data [file qzaf085_supplementary_data.zip › Table S1.docx]

**Table S1 Parameter selection on different datasets**

| **Dataset** | **Sequencing technology** | **K-neighbors** | **α for GCL loss** | **Spot arrangement** | **Resolution** |
| --- | --- | --- | --- | --- | --- |
| DLPFC | 10X Visium | 5 | 0.5 | Hexagon | 220 nm |
| Mouse olfactory bulb | Stereo-seq | 10 | 0.5 | Square | single cell |
|  | Spatial transcriptomics | 3 | 0.5 | Square | 100 μm |
| Mouse brain serial | 10X Visium | 5 | 0.5 | Hexagon | 220 nm |
| Mouse organogenesis | Stereo-seq | 10 | 10 | Square | single cell |
|  | seqFISH | 8 | 10 | Irregular | single cell |
| Human breast cancer | 10X Visium | 5 | 0.5 | Hexagon | 220 nm |

*Note*: The selection of the number of neighbors to build spatial adjacency graph is directly related to spot arrangement and resolution. DLPFC, dorsolateral prefrontal cortex; GCL, graph contrastive learning; Stereo-seq, spatial enhanced resolution omics sequencing; seqFISH, sequential fluorescence in situ hybridization.
