# Supplementary material for: DisConST: Distribution-aware Contrastive Learning for Spatial Domain Identification
Source: Genomics Proteomics Bioinformatics. 2025 Sep 24;24(1):qzaf085. doi: 10.1093/gpbjnl/qzaf085 (PMC13317986; doi:10.1093/gpbjnl/qzaf085)
Supplement: qzaf085_Supplementary_Data [file qzaf085_supplementary_data.zip › Table S2.docx]

**Table S2 The state-of-the-art methods for spatial domain identification in spatial transcriptomics**

| **Method** | **Clustering method** | **Use histology image** | **Source code link** |
| --- | --- | --- | --- |
| stLearn | K-means | Yes | https://github.com/BiomedicalMachineLearning/stLearn |
| SEDR | Leiden | No | https://github.com/JinmiaoChenLab/SEDR |
| SpaGCN | DEC | Yes | https://github.com/jianhuupenn/SpaGCN |
| CCST | K-means | No | https://github.com/xiaoyeye/CCST |
| BayesSpace | mclust | No | <https://github.com/edward130603/BayesSpace> |
| STAGATE | mclust | No | https://github.com/QIFEIDKN/STAGATE_pyG |
| GraphST | mclust | No | https://github.com/JinmiaoChenLab/GraphST |

*Note*: DEC, deep embedded clustering.
