# Supplementary material for: DisConST: Distribution-aware Contrastive Learning for Spatial Domain Identification
Source: Genomics Proteomics Bioinformatics. 2025 Sep 24;24(1):qzaf085. doi: 10.1093/gpbjnl/qzaf085 (PMC13317986; doi:10.1093/gpbjnl/qzaf085)
Supplement: qzaf085_Supplementary_Data [file qzaf085_supplementary_data.zip › Table S4.docx]

**Table S2 ARI scores of DisConST and seven comparison methods on 12 DLPFC slices**

| **Slice** | **stLearn** | **SEDR** | **SpaGCN** | **CCST** | **BayesSpace** | **STAGATE** | **GraphST** | **DisConST (l)** | **DisConST (k)** | **DisConST (m)** |
| --- | --- | --- | --- | --- | --- | --- | --- | --- | --- | --- |
| 151507 | 0.4556 | 0.4040 | 0.3798 | 0.4833 | 0.4690 | 0.5347 | 0.4575 | 0.5699 | 0.5825 | **0.5977** |
| 151508 | 0.2948 | 0.2560 | 0.4426 | 0.3097 | 0.4369 | 0.4919 | 0.4656 | 0.4604 | 0.5103 | **0.5531** |
| 151509 | 0.4148 | 0.2884 | 0.4566 | 0.4027 | 0.3814 | 0.4837 | 0.4746 | 0.5222 | **0.5636** | 0.5578 |
| 151510 | 0.2703 | 0.2867 | 0.4524 | 0.4332 | 0.3767 | 0.4800 | 0.4780 | 0.4549 | 0.4996 | **0.5763** |
| 151669 | 0.3399 | 0.2136 | 0.2920 | 0.2627 | 0.4704 | 0.2565 | 0.3983 | 0.5374 | 0.5032 | **0.5862** |
| 151670 | 0.1871 | 0.2033 | 0.3395 | 0.2810 | 0.4291 | 0.3902 | 0.4023 | 0.4420 | 0.4671 | **0.5611** |
| 151671 | 0.2810 | 0.4238 | 0.5268 | 0.6545 | 0.7334 | 0.5855 | 0.5835 | 0.6390 | 0.6047 | **0.8569** |
| 151672 | 0.3441 | 0.5013 | 0.5394 | 0.6217 | 0.4389 | 0.5994 | 0.5978 | 0.5695 | 0.6041 | **0.7639** |
| 151673 | 0.3133 | 0.4507 | 0.4513 | 0.5638 | 0.5499 | 0.6047 | 0.6164 | 0.5363 | 0.5415 | **0.6183** |
| 151674 | 0.3205 | 0.3613 | 0.4390 | 0.4003 | 0.2959 | 0.4407 | 0.5812 | 0.5067 | 0.5459 | **0.6542** |
| 151675 | 0.4502 | 0.4957 | 0.3152 | 0.3700 | 0.5297 | 0.5841 | 0.5362 | 0.4591 | 0.5080 | **0.5636** |
| 151676 | 0.3588 | 0.4654 | 0.3984 | 0.4016 | 0.3642 | 0.5518 | 0.5383 | 0.5379 | 0.5276 | **0.6070** |
| Upper quartile | 0.3728 | 0.4544 | 0.4535 | 0.5034 | 0.4852 | 0.5844 | 0.5818 | 0.5458 | 0.5683 | **0.6273** |
| Median | 0.3302 | 0.3827 | 0.4408 | 0.4022 | 0.4379 | 0.5133 | 0.5071 | 0.5293 | 0.5346 | **0.5920** |
| Lower quartile | 0.2914 | 0.2790 | 0.3697 | 0.3549 | 0.3802 | 0.4702 | 0.4636 | 0.4601 | 0.5068 | **0.5630** |
| Average | 0.3359 | 0.3625 | 0.4192 | 0.4320 | 0.4563 | 0.5003 | 0.5108 | 0.5196 | 0.5382 | **0.6247** |

*Note*: (k)/(l)/(m) represent K-means, Leiden, and mclust clustering methods, respectively. ARI, Adjusted Rand Index; SEDR, Spatially Embedded Deep Representation; CCST, Cell Clustering for Spatial Transcriptomics; DisConST, Distribution-aware Contrastive Learning for Spatial Transcriptomics. Bold represents the best method on the data.
