# Supplementary material for: DisConST: Distribution-aware Contrastive Learning for Spatial Domain Identification
Source: Genomics Proteomics Bioinformatics. 2025 Sep 24;24(1):qzaf085. doi: 10.1093/gpbjnl/qzaf085 (PMC13317986; doi:10.1093/gpbjnl/qzaf085)
Supplement: qzaf085_Supplementary_Data [file qzaf085_supplementary_data.zip › Table S5.docx]

**Table S5 AMI scores of DisConST and seven comparison methods on 12 DLPFC slices**

| **Slice** | **stLearn** | **SEDR** | **SpaGCN** | **CCST** | **BayesSpace** | **STAGATE** | **GraphST** | **DisConST (l)** | **DisConST (k)** | **DisConST (m)** |
| --- | --- | --- | --- | --- | --- | --- | --- | --- | --- | --- |
| 151507 | 0.5952 | 0.5228 | 0.5108 | 0.6433 | 0.6270 | 0.7035 | 0.6657 | 0.6850 | 0.7038 | **0.7178** |
| 151508 | 0.5138 | 0.4690 | 0.5376 | 0.5290 | 0.5981 | 0.6211 | 0.5249 | 0.6152 | 0.6460 | **0.6811** |
| 151509 | 0.6050 | 0.5162 | 0.5511 | 0.6078 | 0.5915 | 0.6466 | 0.6443 | 0.6862 | **0.7066** | 0.6823 |
| 151510 | 0.4986 | 0.4729 | 0.5689 | 0.5895 | 0.5519 | 0.6510 | 0.6449 | 0.6235 | 0.6337 | **0.6646** |
| 151669 | 0.5137 | 0.4907 | 0.4518 | 0.4571 | 0.6092 | 0.6227 | 0.5522 | **0.6340** | 0.6119 | 0.6307 |
| 151670 | 0.3541 | 0.4289 | 0.4463 | 0.4364 | 0.5538 | 0.5638 | 0.6219 | 0.5639 | 0.5891 | **0.6646** |
| 151671 | 0.4613 | 0.5507 | 0.6292 | 0.6397 | 0.6885 | 0.7074 | 0.6443 | 0.7423 | 0.7350 | **0.8097** |
| 151672 | 0.4853 | 0.5659 | 0.6485 | 0.6420 | 0.5957 | 0.6895 | 0.5959 | 0.6639 | 0.6942 | **0.7156** |
| 151673 | 0.4969 | 0.6377 | 0.5248 | 0.6566 | 0.6872 | 0.7142 | 0.7161 | 0.6830 | 0.6847 | **0.7165** |
| 151674 | 0.5000 | 0.5424 | 0.4681 | 0.6138 | 0.4804 | 0.6206 | 0.6607 | 0.6555 | 0.6937 | **0.7502** |
| 151675 | 0.5809 | 0.6062 | 0.4564 | 0.5722 | **0.6839** | **0.6839** | 0.5799 | 0.6200 | 0.6622 | 0.6706 |
| 151676 | 0.5323 | 0.6096 | 0.4768 | 0.6014 | 0.5582 | 0.5982 | 0.6035 | 0.6522 | 0.6639 | **0.7031** |
| Average | 0.5114 | 0.5344 | 0.5225 | 0.5824 | 0.6021 | 0.6519 | 0.6212 | 0.6521 | 0.6687 | **0.7006** |

*Note*: (k)/(l)/(m) represent K-means, Leiden, and mclust clustering methods, respectively. AMI, Adjusted Mutual Information. Bold represents the best method on the data.
