# Supplementary material for: DisConST: Distribution-aware Contrastive Learning for Spatial Domain Identification
Source: Genomics Proteomics Bioinformatics. 2025 Sep 24;24(1):qzaf085. doi: 10.1093/gpbjnl/qzaf085 (PMC13317986; doi:10.1093/gpbjnl/qzaf085)
Supplement: qzaf085_Supplementary_Data [file qzaf085_supplementary_data.zip › Table S6.docx]

**Table S6 NMI scores of DisConST and seven comparison methods on 12 DLPFC slices**

| **Slice** | **stLearn** | **SEDR** | **SpaGCN** | **CCST** | **BayesSpace** | **STAGATE** | **GraphST** | **DisConST (l)** | **DisConST (k)** | **DisConST (m)** |
| --- | --- | --- | --- | --- | --- | --- | --- | --- | --- | --- |
| 151507 | 0.5962 | 0.5243 | 0.5119 | 0.6442 | 0.6279 | 0.7042 | 0.6665 | 0.6859 | 0.7046 | **0.7186** |
| 151508 | 0.5150 | 0.4704 | 0.5387 | 0.5302 | 0.5991 | 0.6218 | 0.5261 | 0.6162 | 0.6469 | **0.6820** |
| 151509 | 0.6059 | 0.5174 | 0.5521 | 0.6087 | 0.5924 | 0.6474 | 0.6451 | 0.6870 | **0.7073** | 0.6832 |
| 151510 | 0.4998 | 0.4742 | 0.5698 | 0.5905 | 0.5530 | 0.6518 | 0.6458 | 0.6245 | 0.6347 | **0.6654** |
| 151669 | 0.5145 | 0.4920 | 0.4526 | 0.4578 | 0.6099 | 0.6233 | 0.5528 | 0.6348 | 0.6127 | **0.6388** |
| 151670 | 0.3553 | 0.4307 | 0.4473 | 0.4374 | 0.5546 | 0.5646 | 0.6226 | 0.5647 | 0.5901 | **0.6654** |
| 151671 | 0.4621 | 0.5517 | 0.6297 | 0.6403 | 0.6889 | 0.7078 | 0.6447 | 0.7427 | 0.7355 | **0.8099** |
| 151672 | 0.4861 | 0.5669 | 0.6470 | 0.6425 | 0.5963 | 0.6899 | 0.5964 | 0.6645 | 0.6947 | **0.7161** |
| 151673 | 0.4984 | 0.6390 | 0.5261 | 0.6577 | 0.6880 | 0.7150 | 0.7168 | 0.6840 | 0.6857 | **0.7173** |
| 151674 | 0.5014 | 0.5443 | 0.4695 | 0.6150 | 0.4818 | 0.6216 | 0.6616 | 0.6565 | 0.6947 | **0.7508** |
| 151675 | 0.5820 | 0.6075 | 0.4579 | 0.5733 | 0.6847 | **0.6848** | 0.5809 | 0.6212 | 0.6633 | 0.6715 |
| 151676 | 0.5339 | 0.6112 | 0.4785 | 0.6026 | 0.5596 | 0.5995 | 0.6046 | 0.6533 | 0.6650 | **0.7040** |
| Average | 0.5125 | 0.5358 | 0.5234 | 0.5834 | 0.6030 | 0.6526 | 0.6220 | 0.6529 | 0.6696 | **0.7019** |

*Note*: (k)/(l)/(m) represent K-means, Leiden, and mclust clustering methods, respectively. NMI, Normalized Mutual Information. Bold represents the best method on the data.
