# Supplementary material for: DisConST: Distribution-aware Contrastive Learning for Spatial Domain Identification
Source: Genomics Proteomics Bioinformatics. 2025 Sep 24;24(1):qzaf085. doi: 10.1093/gpbjnl/qzaf085 (PMC13317986; doi:10.1093/gpbjnl/qzaf085)
Supplement: qzaf085_Supplementary_Data [file qzaf085_supplementary_data.zip › Table S7.docx]

**Table S7 ARI scores of DisConST and seven comparison methods on all datasets**

| **Dataset** | **stLearn** | **SEDR** | **SpaGCN** | **CCST** | **BayesSpace** | **STAGATE** | **GraphST** | **DisConST** |
| --- | --- | --- | --- | --- | --- | --- | --- | --- |
| Mouse olfactory bulb (Stereo-seq) | NA | 0.4746 | 0.5535 | 0.5759 | NA | 0.4795 | 0.2693 | **0.5789** |
| Mouse olfactory bulb (Spatial transcriptomics) | NA | 0.0336 | 0.5380 | 0.0172 | NA | 0.3435 | 0.5038 | **0.5460** |
| Mouse organogenesis  (seqFISH) | NA | NA | 0.3857 | 0.2575 | NA | 0.1143 | 0.3472 | **0.4000** |
| Mouse brain serial (Anterior) | 0.4836 | 0.5038 | 0.5488 | 0.4595 | 0.5318 | 0.4578 | 0.5613 | **0.5837** |
| Mouse brain serial (Posterior) | 0.5023 | 0.4817 | 0.5890 | 0.4955 | **0.6825** | 0.5333 | 0.5253 | 0.6125 |
| Mouse organogenesis (Stereo-seq E9.5) | NA | 0.3826 | 0.4976 | 0.4288 | NA | 0.4204 | 0.4161 | **0.5104** |
| Mouse organogenesis (Stereo-seq E10.5) | NA | 0.3468 | **0.3879** | 0.2748 | NA | 0.2375 | 0.2614 | 0.3566 |
| Mouse organogenesis (Stereo-seq E11.5) | NA | NA | 0.3506 | 0.2700 | NA | **0.3582** | 0.2676 | 0.3569 |
| Mouse organogenesis (Stereo-seq E12.5) | NA | NA | 0.3639 | 0.3647 | NA | 0.4076 | 0.3539 | **0.3866** |
| Human breast cancer | 0.5717 | 0.5090 | 0.5017 | 0.5523 | 0.5576 | 0.4423 | 0.4936 | **0.6201** |

*Note*: E9.5, embryonic day 9.5.
