# Supplementary figures and images for: DisConST: Distribution-aware Contrastive Learning for Spatial Domain Identification
Source: Genomics Proteomics Bioinformatics. 2025 Sep 24;24(1):qzaf085. doi: 10.1093/gpbjnl/qzaf085 (PMC13317986; doi:10.1093/gpbjnl/qzaf085)

A

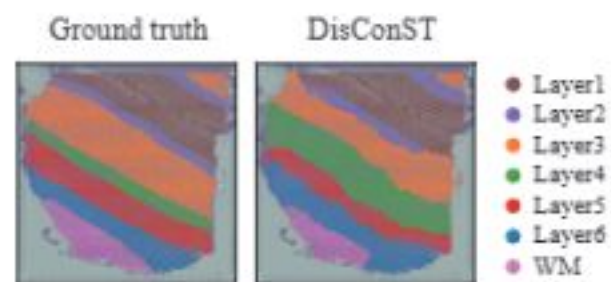

B

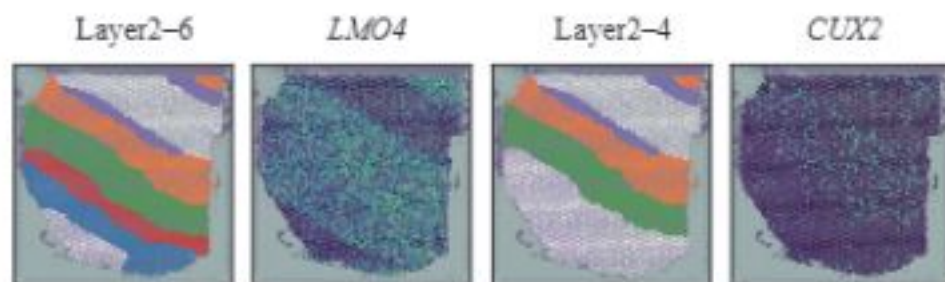

C

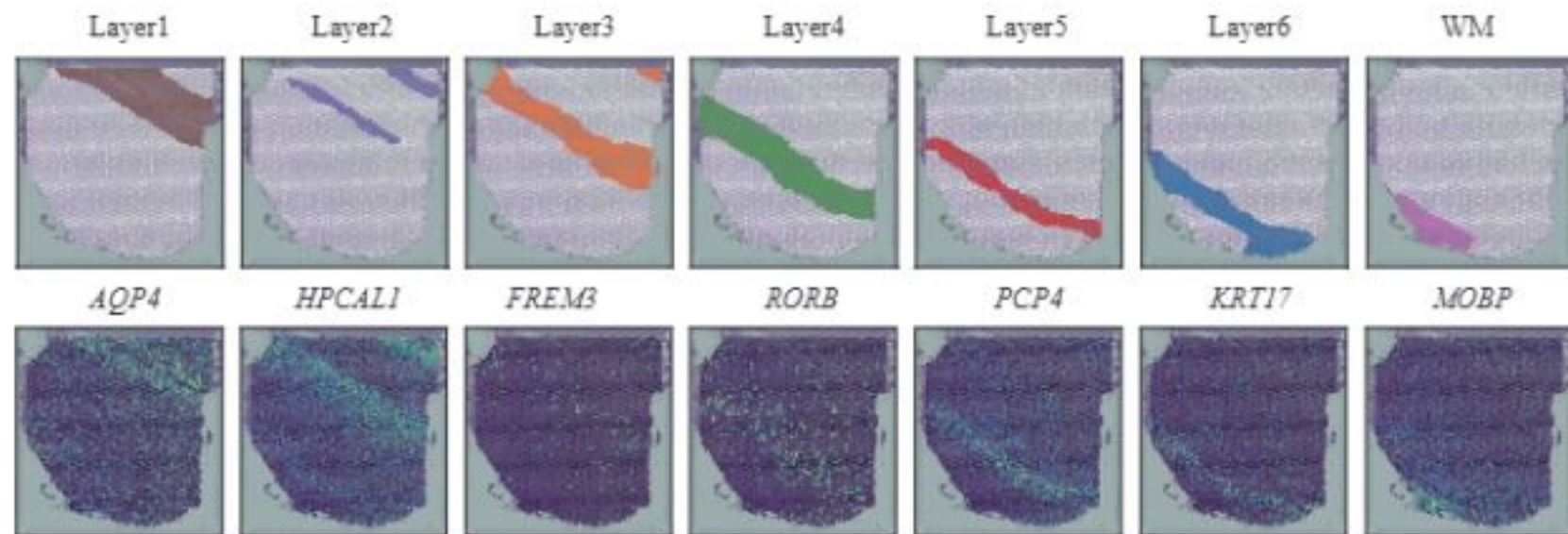

Supplement: qzaf085_Supplementary_Data [file qzaf085_supplementary_data.zip › Figure S1 (1).pdf]

average ARI = 0.3559

STAGATE

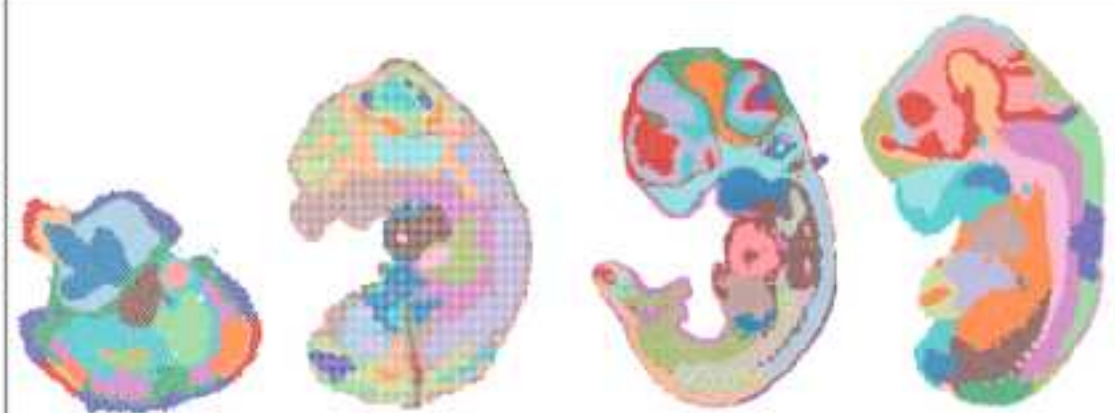

average ARI = 0.3247

GraphST

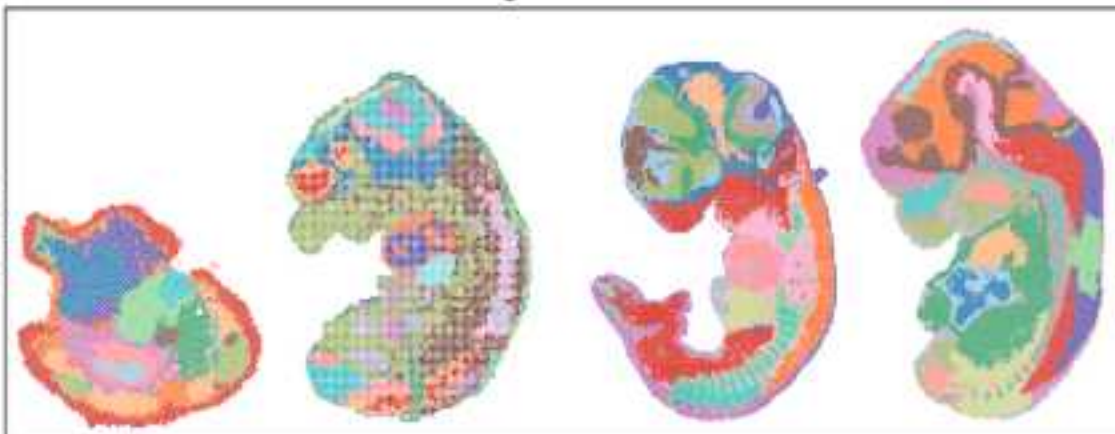

average ARI = 0.3346

CCST

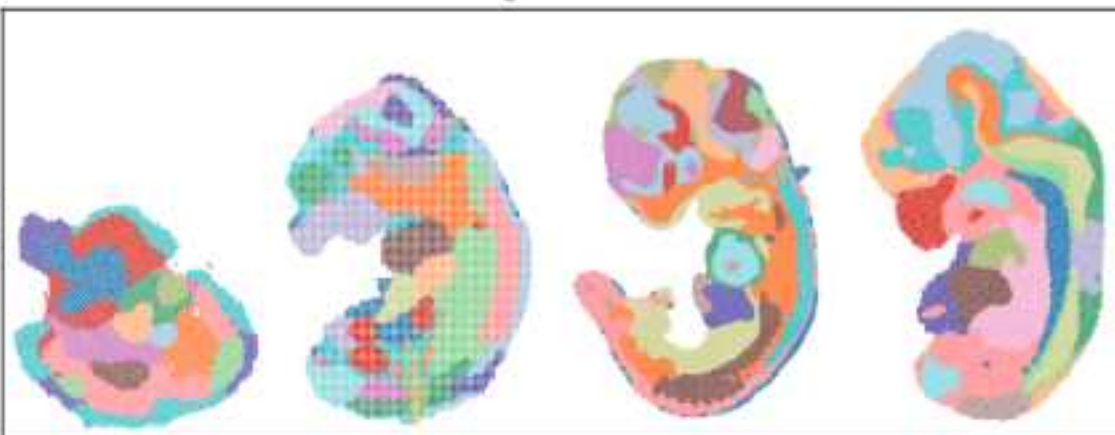

average ARI = 0.3999

SpaGCN

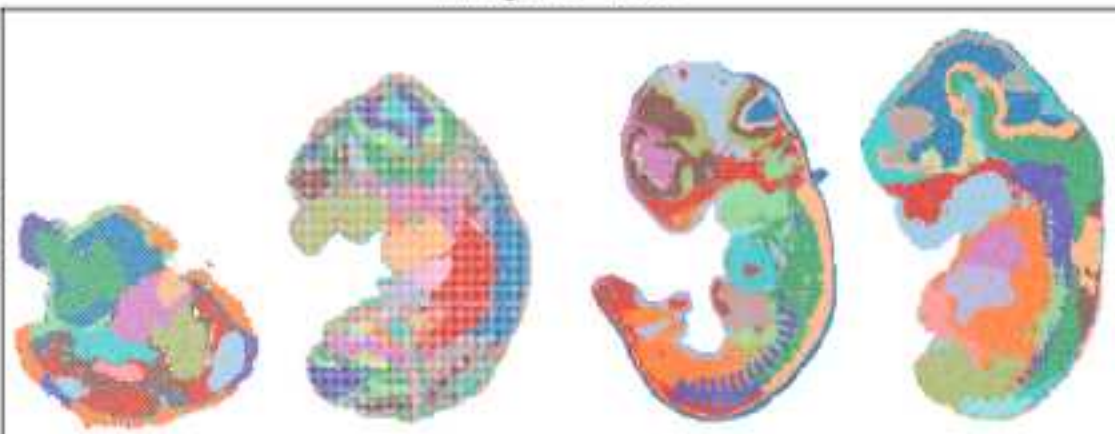

average ARI = 0.3647

SEDR

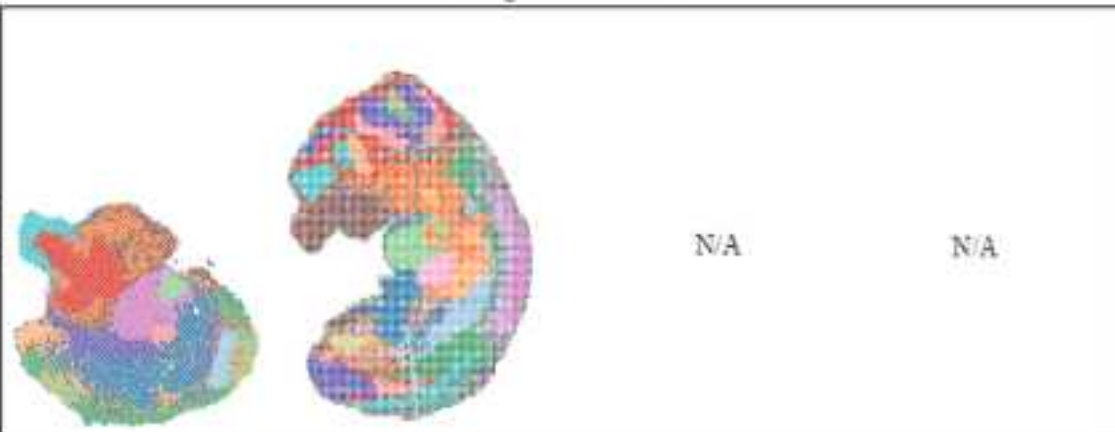

Supplement: qzaf085_Supplementary_Data [file qzaf085_supplementary_data.zip › Figure S10 (1).pdf]

stLearn ARI = 0.5717

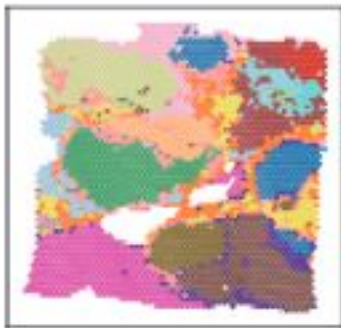

SEDR ARI = 0.5090

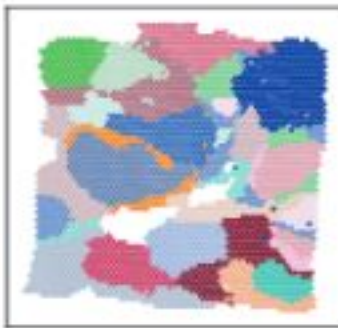

SpaGCN ARI = 0.5017

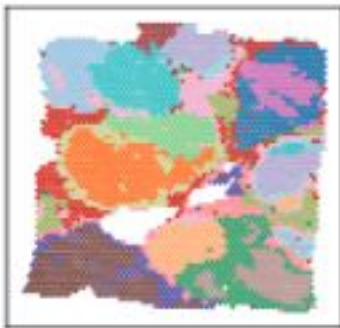

CCST ARI = 0.5523

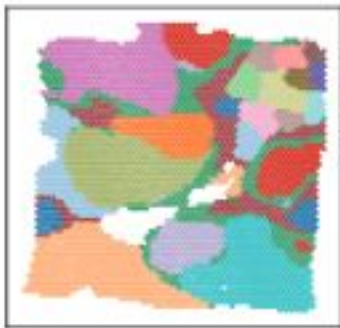

BayesSpace ARI = 0.5576

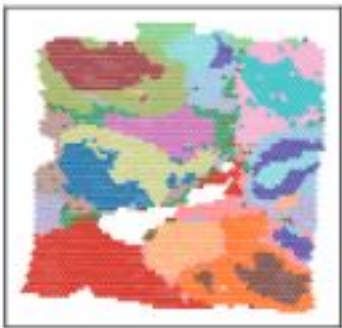

Supplement: qzaf085_Supplementary_Data [file qzaf085_supplementary_data.zip › Figure S13 (1).pdf]

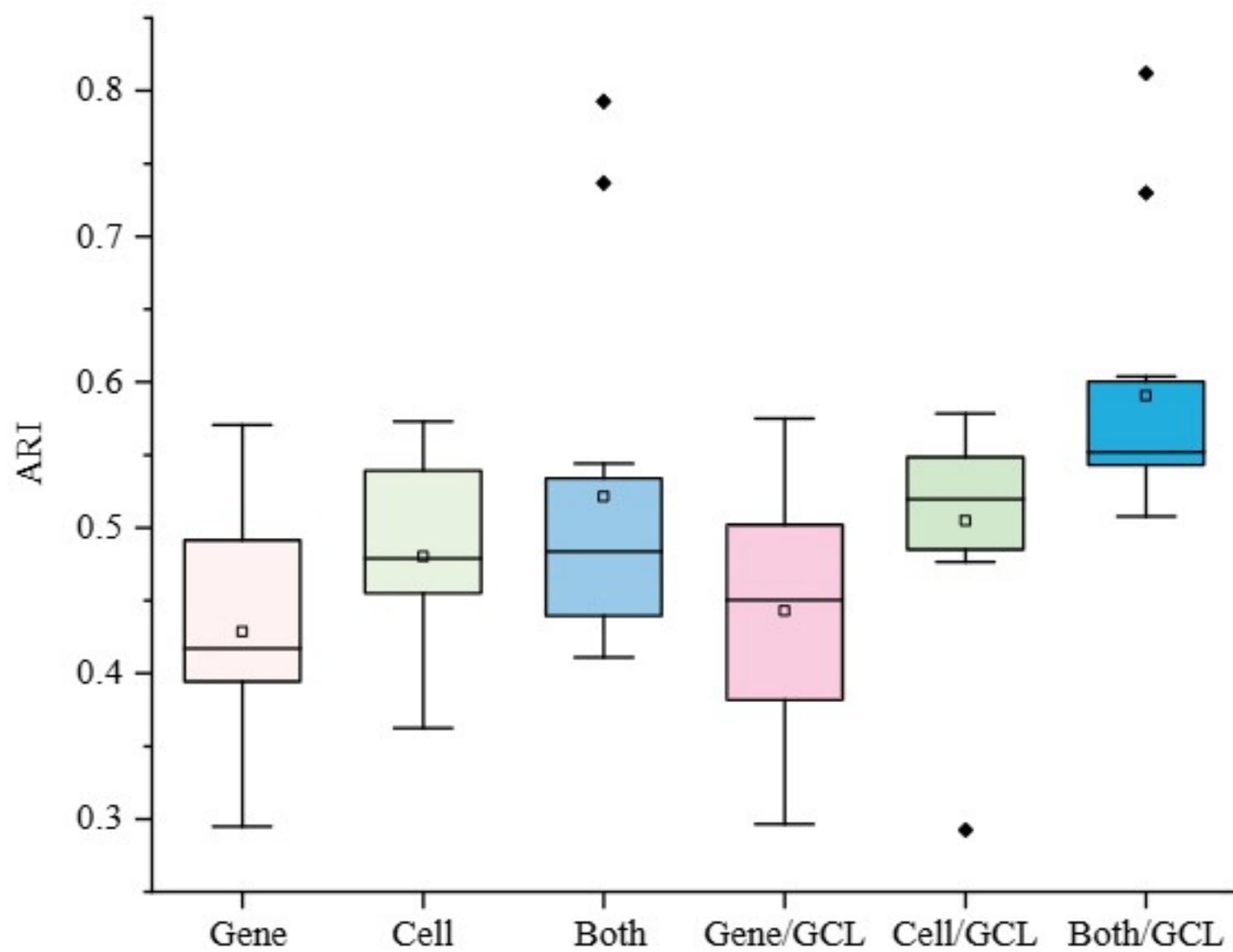

Supplement: qzaf085_Supplementary_Data [file qzaf085_supplementary_data.zip › Figure S15 (1).pdf]

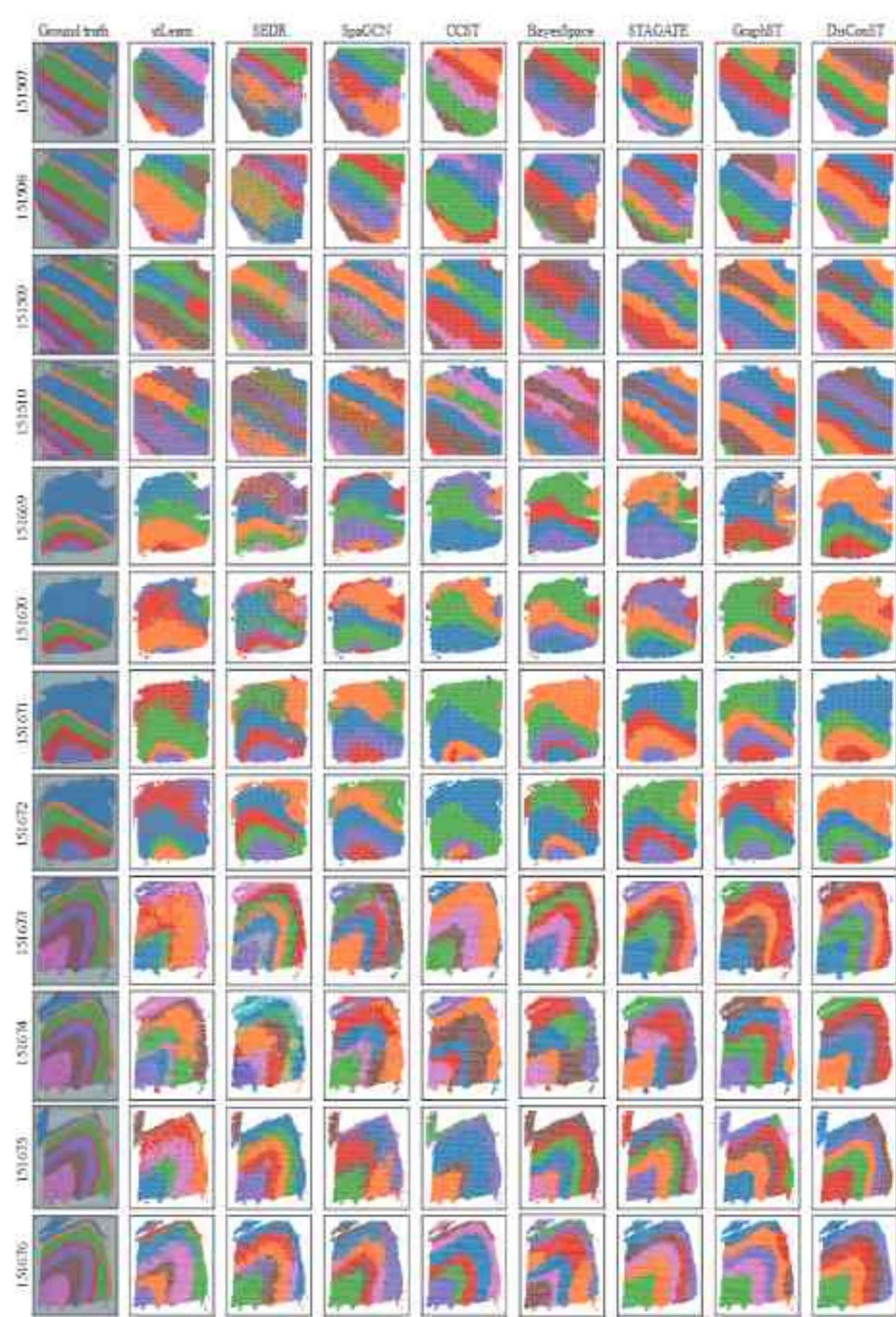

Supplement: qzaf085_Supplementary_Data [file qzaf085_supplementary_data.zip › Figure S2 (1).pdf]

A

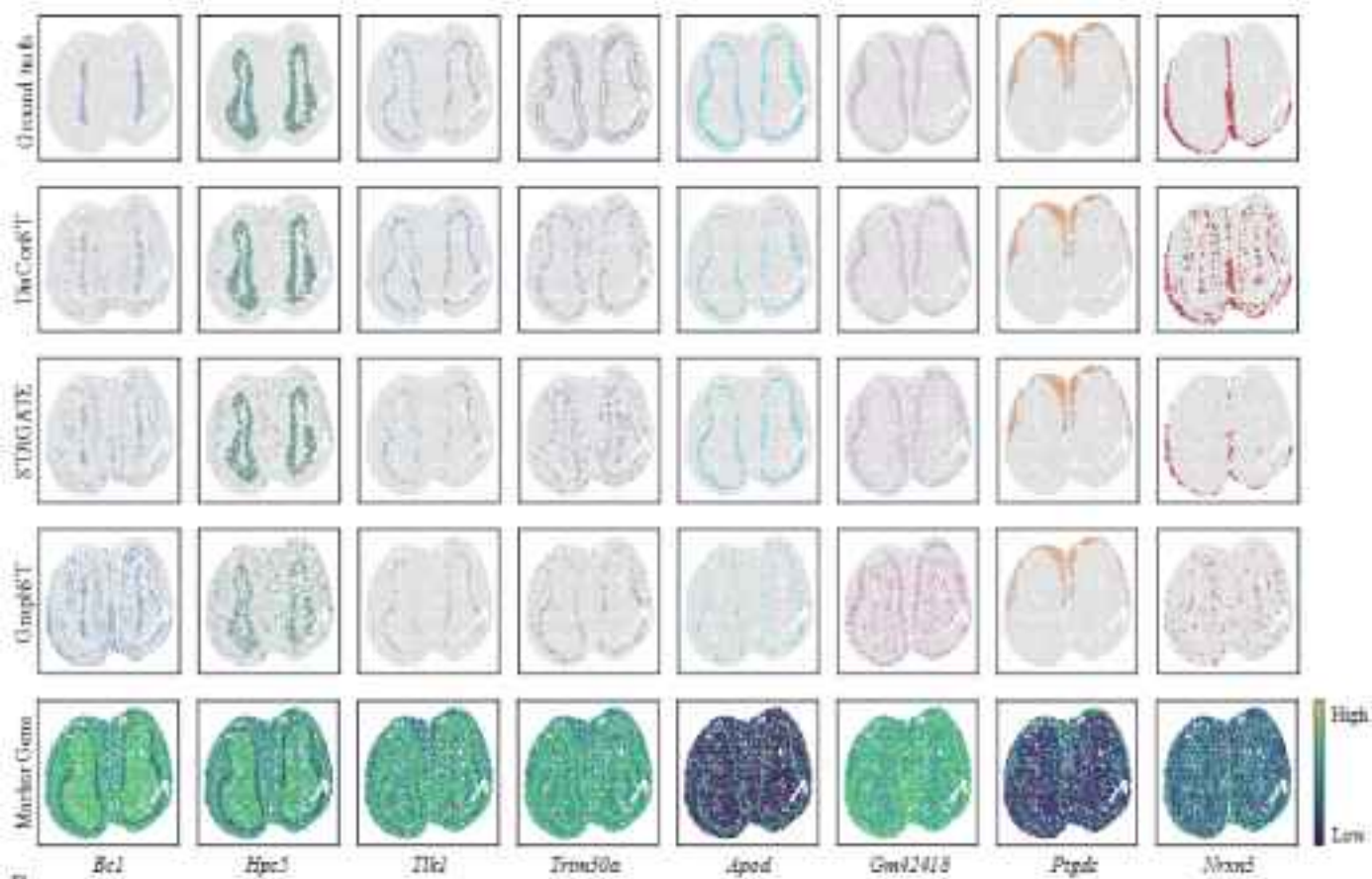

B

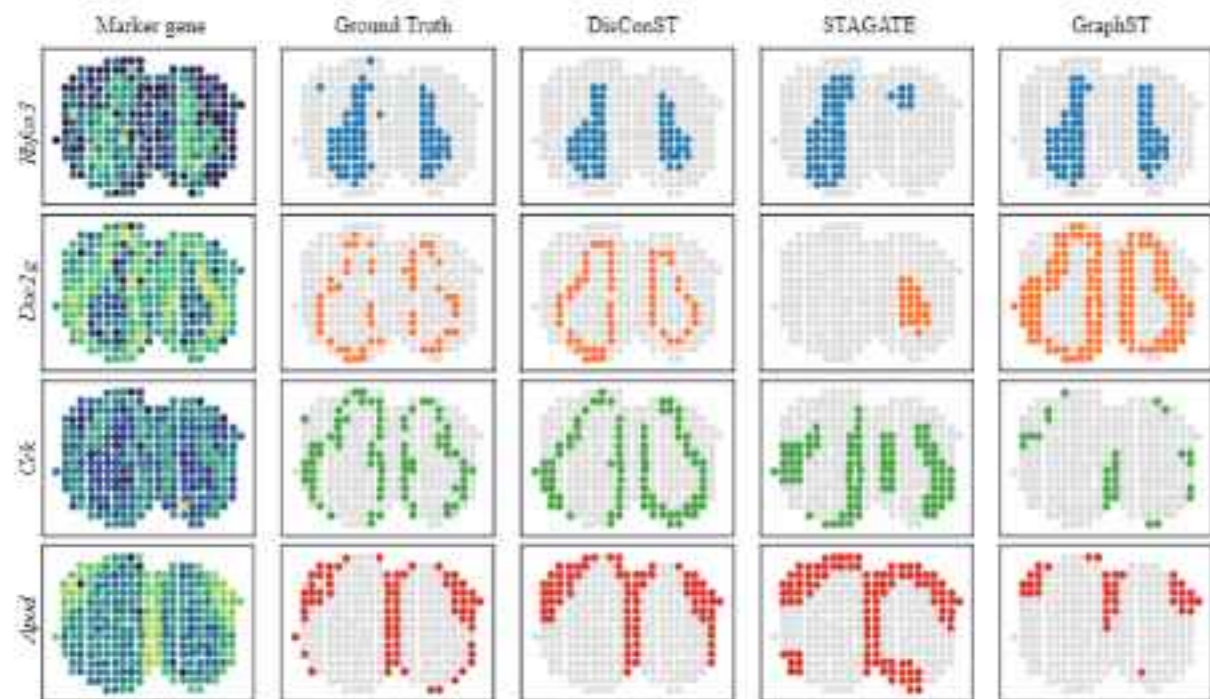

Supplement: qzaf085_Supplementary_Data [file qzaf085_supplementary_data.zip › Figure S3 (1).pdf]

SEDR

ARI = 0.4746

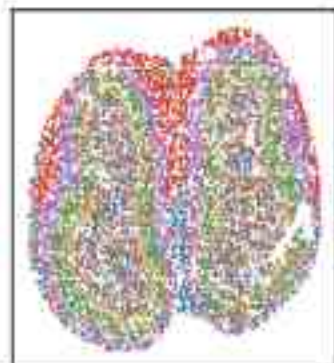

ARI = 0.0336

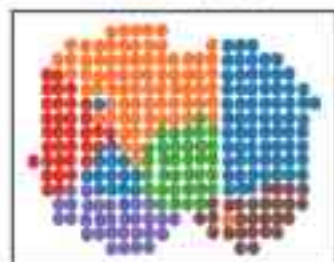

N/A

ARI = 0.3826

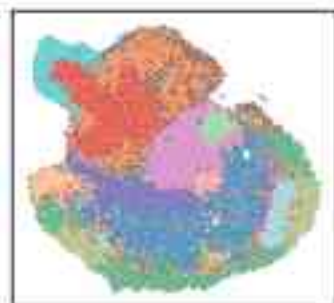

SpaGCN

ARI = 0.5535

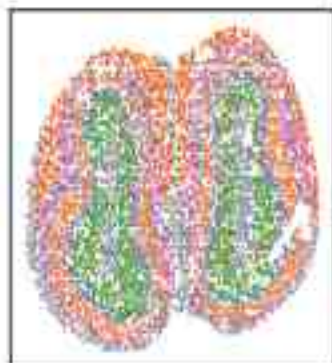

ARI = 0.5380

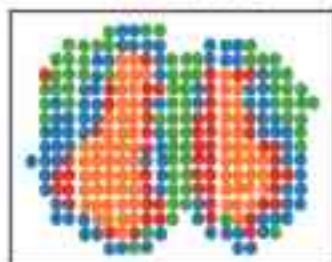

ARI = 0.3857

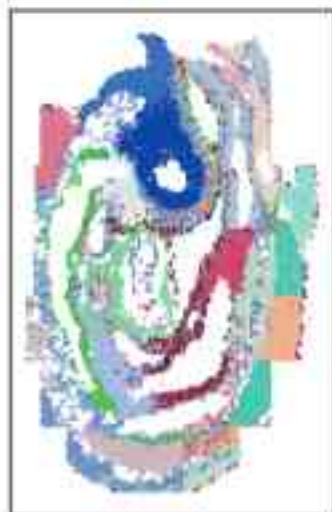

ARI = 0.4976

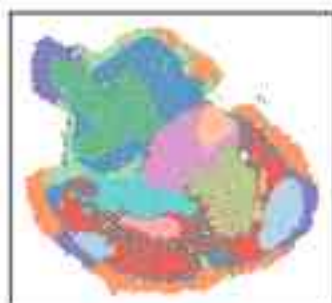

CCST

ARI = 0.5759

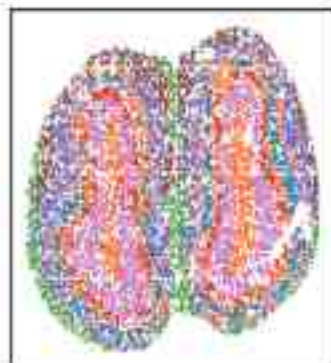

ARI = 0.0172

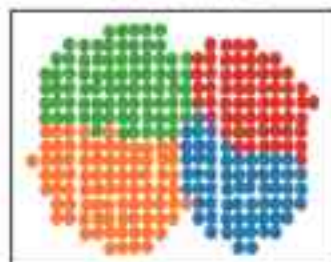

ARI = 0.2575

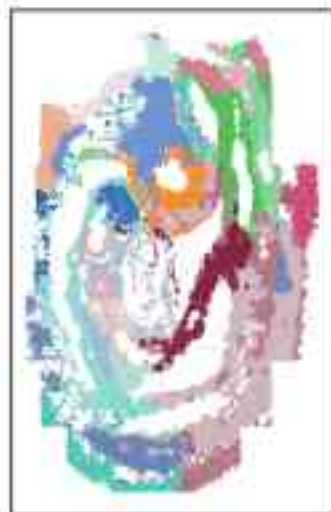

ARI = 0.4288

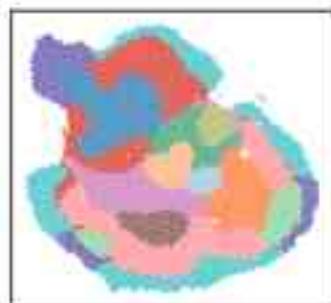

Supplement: qzaf085_Supplementary_Data [file qzaf085_supplementary_data.zip › Figure S4 (1).pdf]

stLearn

ARI = 0.4836

ARI = 0.5023

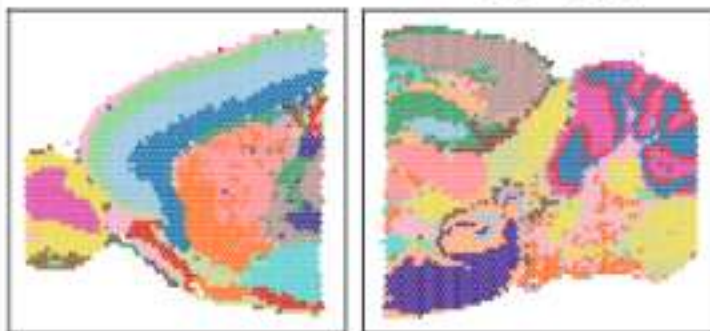

SEDR

ARI = 0.5038

ARI = 0.4817

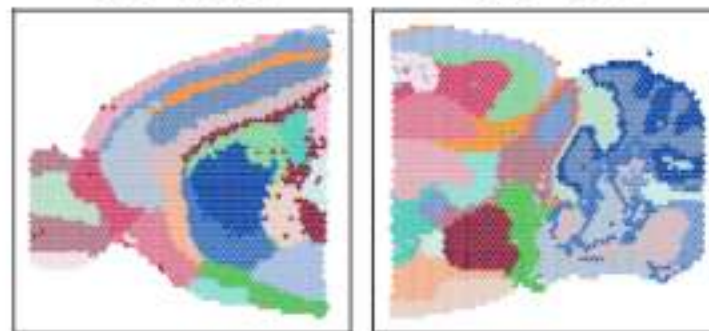

SpaGCN

ARI = 0.5488

ARI = 0.5890

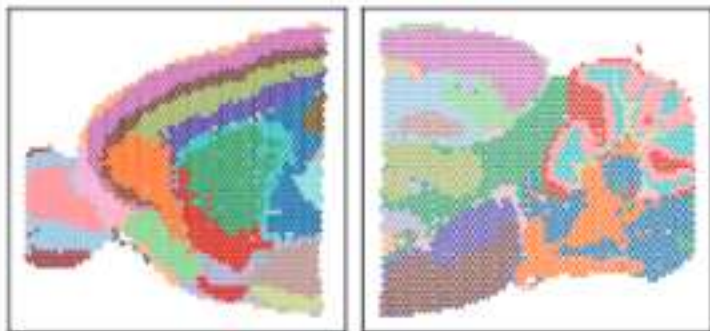

CCST

ARI = 0.4595

ARI = 0.4955

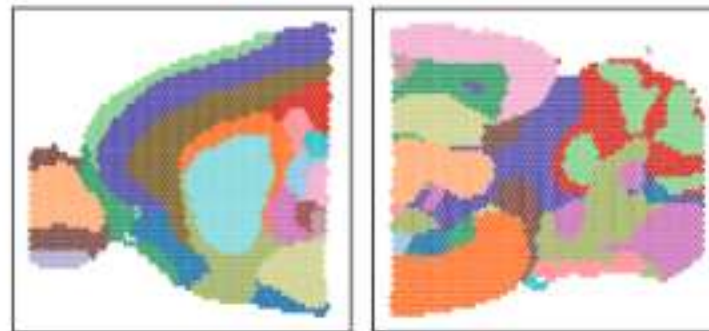

BayesSpace

ARI = 0.5318

ARI = 0.6825

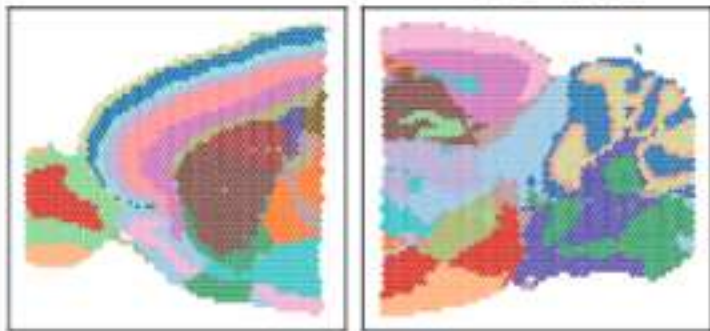

Supplement: qzaf085_Supplementary_Data [file qzaf085_supplementary_data.zip › Figure S5 (1).pdf]

A

Histology image

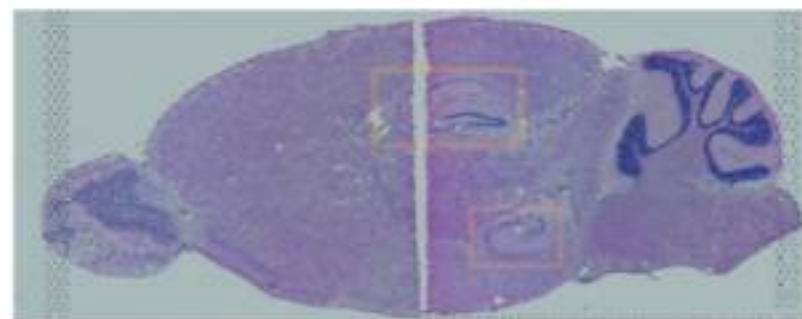

B

Allen Brain Atlas

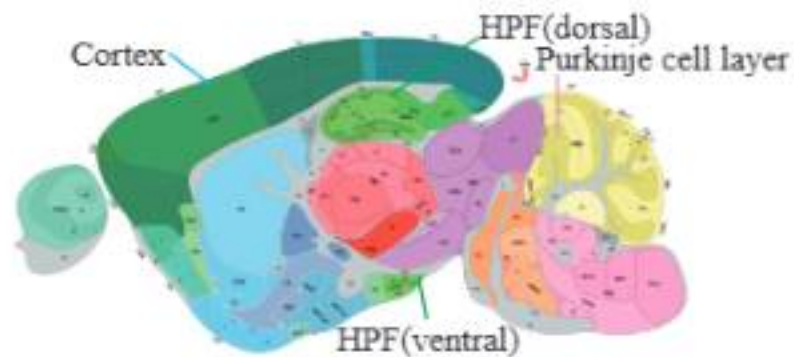

C

Spatial clustering

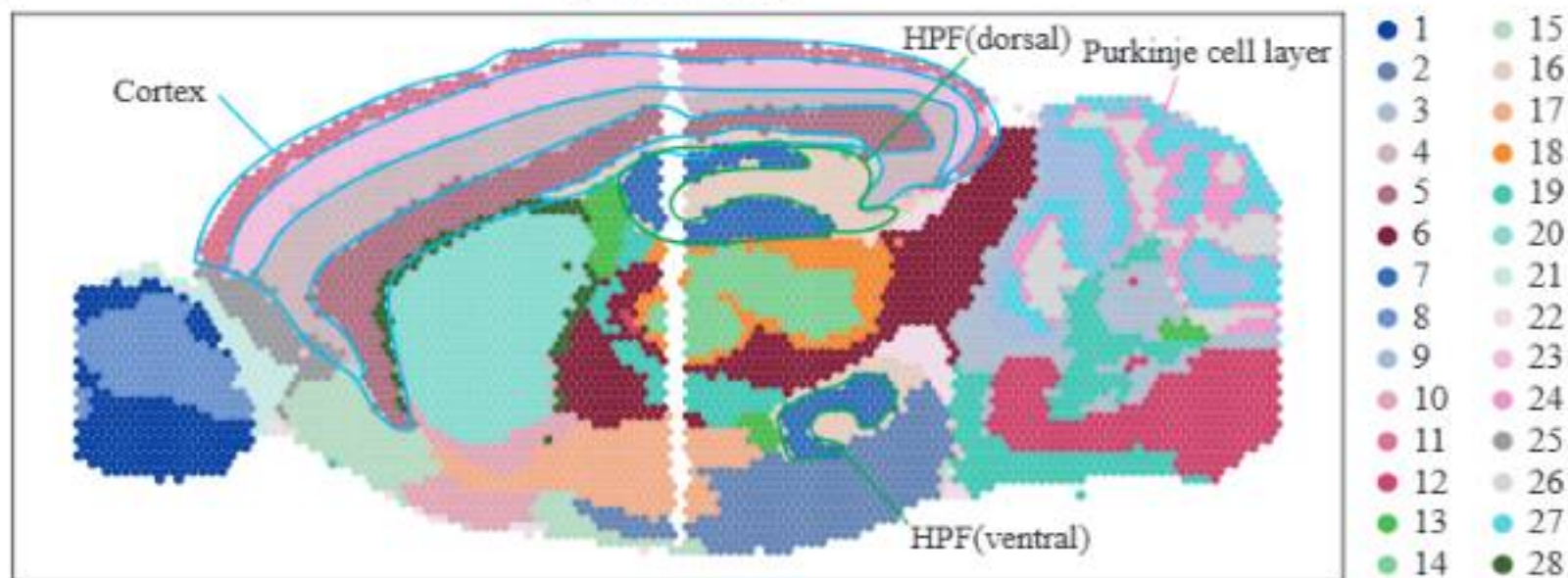

Supplement: qzaf085_Supplementary_Data [file qzaf085_supplementary_data.zip › Figure S7 (1).pdf]

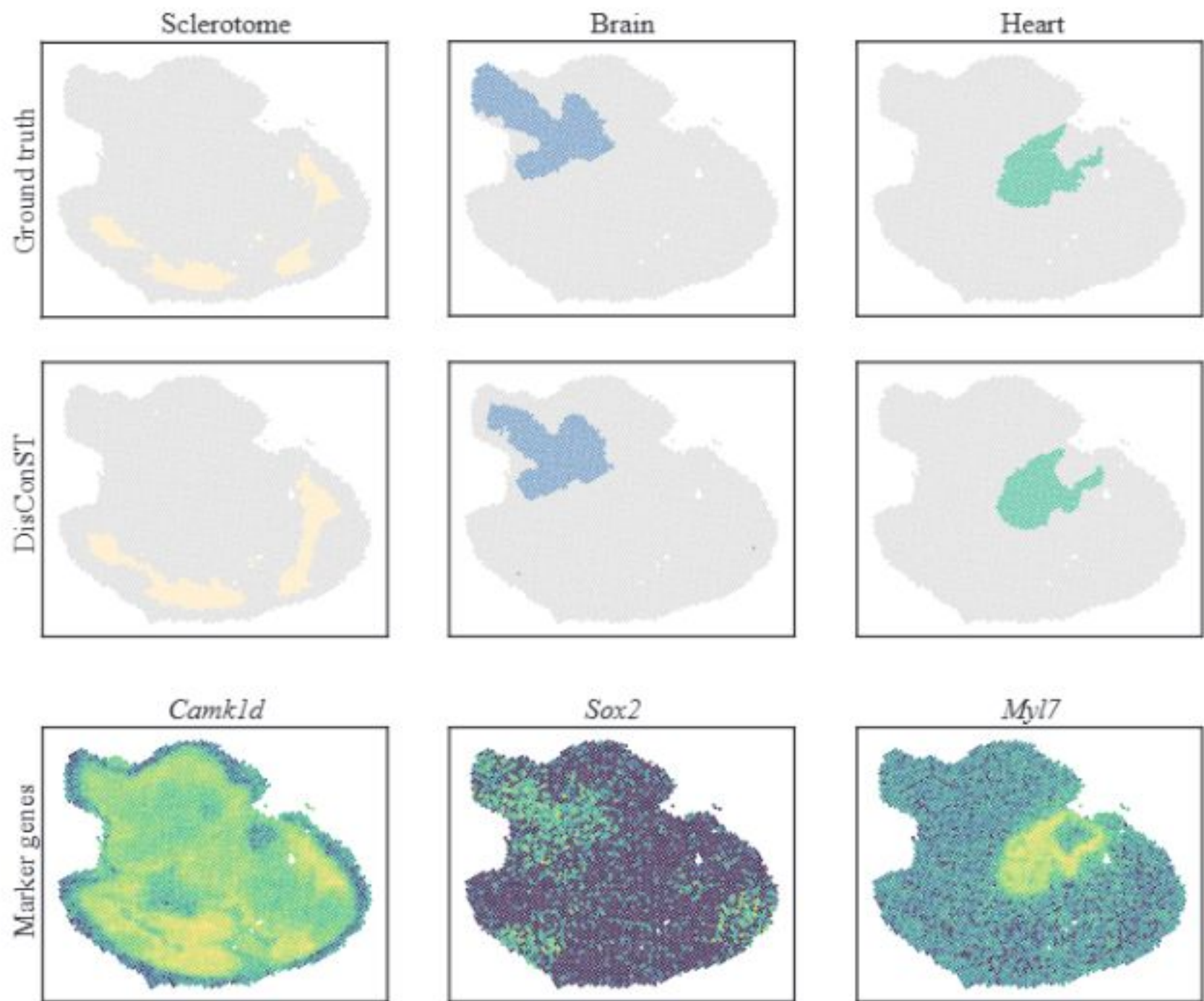

Supplement: qzaf085_Supplementary_Data [file qzaf085_supplementary_data.zip › Figure S8 (1).pdf]

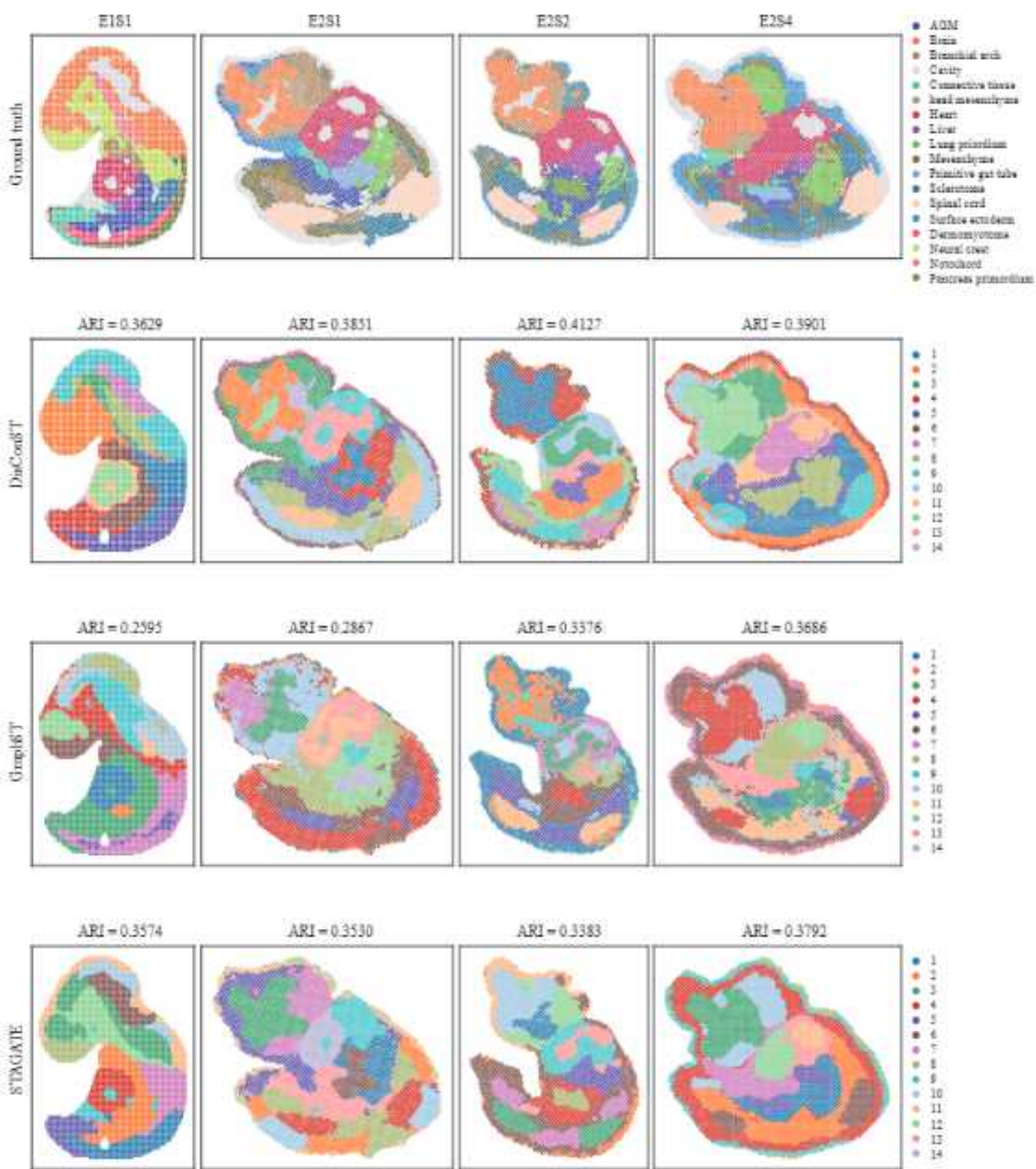

Supplement: qzaf085_Supplementary_Data [file qzaf085_supplementary_data.zip › Figure S9 (1).pdf]
